# Exploring the RNase A scaffold to combine catalytic and antimicrobial activities. Structural characterization of RNase 3/1 chimeras

**DOI:** 10.3389/fmolb.2022.964717

**Published:** 2022-09-14

**Authors:** Pablo Fernández-Millán, Sergi Vázquez-Monteagudo, Ester Boix, Guillem Prats-Ejarque

**Affiliations:** Department of Biochemistry and Molecular Biology, Faculty of Biosciences, Universitat Autònoma de Barcelona, Cerdanyola del Vallès, Spain

**Keywords:** RNase 3, RNase 1, chimera, crystal structure, catalytic activity, base binding sites, antimicrobial protein

## Abstract

Design of novel antibiotics to fight antimicrobial resistance is one of the first global health priorities. Novel protein-based strategies come out as alternative therapies. Based on the structure-function knowledge of the RNase A superfamily we have engineered a chimera that combines RNase 1 highest catalytic activity with RNase 3 unique antipathogen properties. A first construct (RNase 3/1-v1) was successfully designed with a catalytic activity 40-fold higher than RNase 3, but alas in detriment of its anti-pathogenic activity. Next, two new versions of the original chimeric protein were created showing improvement in the antimicrobial activity. Both second generation versions (RNases 3/1-v2 and -v3) incorporated a loop characteristic of RNase 3 (L7), associated to antimicrobial activity. Last, removal of an RNase 1 flexible loop (L1) in the third version enhanced its antimicrobial properties and catalytic efficiency. Here we solved the 3D structures of the three chimeras at atomic resolution by X-ray crystallography. Structural analysis outlined the key functional regions. Prediction by molecular docking of the protein chimera in complex with dinucleotides highlighted the contribution of the C-terminal region to shape the substrate binding cavity and determine the base selectivity and catalytic efficiency. Nonetheless, the structures that incorporated the key features related to RNase 3 antimicrobial activity retained the overall RNase 1 active site conformation together with the essential structural elements for binding to the human ribonuclease inhibitor (RNHI), ensuring non-cytotoxicity. Results will guide us in the design of the best RNase pharmacophore for anti-infective therapies.

## 1 Introduction

There is an urgent need to develop novel antimicrobial agents that explore alternative mechanisms of action. Antibiotics based on antimicrobial proteins and peptides (AMPPs) from our own innate immune system offer attractive advantages, such as reduced toxicity and immunogenicity. Despite the potentially expensive cost of protein-based drugs respect to small molecules, novel methodologies have being developed to minimize the large scale production cost (Gifre-Renom et al., 2018). RNases as cellular RNA metabolizing enzymes are attractive candidates for drug development (Canestrari and Paroo, 2018; Garnett and Raines, 2021). Particular interest was drawn by members of the RNase A superfamily endowed with specific anti-infective properties that complement their catalytic properties (Boix and Nogués, 2007; Rosenberg, 2008; Lu et al., 2018; Li and Boix, 2021). In addition, nanodelivery systems have been tailored for RNase delivery to infected cells (Rangel-Muñoz et al., 2020).

Among the so called “human antimicrobial RNases,” RNase 3 stands out for its unique high isoelectric point (pI ∼11) and its enhanced ability to bind and destabilize microbial envelopes (Boix et al., 2012; Torrent et al., 2012; Lu et al., 2018). Unfortunately, the high antimicrobial activity and cationicity of RNase 3 was positively selected during primate evolution in detriment of its enzymatic activity (Zhang et al., 1998).

In our laboratory we recently designed an RNase chimera that combines the high catalytic activity of RNase 1 with specific antimicrobial regions of RNase 3. The construct was successfully tested against the emergence of bacterial resistance (Prats-Ejarque et al., 2019a). Following, two new variants were engineered to enhance the antimicrobial activity, while retaining a high catalytic action. The three chimeras were validated by assessing their kinetic and antimicrobial properties (Prats-Ejarque et al., 2021). Special attention was dedicated to ensure conservation of the key interacting residues to the mammalian ribonuclease inhibitor to ensure non-toxicity to host cells (Lomax et al., 2012).

In this work we crystallized the three versions of the RNase 3/1 chimera and solved their three-dimensional structures at atomic resolution. Structural data analysis and molecular modelling enabled us to identify the key determinants that account for the specific properties of each variant.

## 2 Methods

### 2.1 Cloning and expression

The RNases 1, 3 and the three versions of RNase 3/1 genes were subcloned into the plasmid pET11c for prokaryote high yield expression in *E. coli* BL21 (DE3) strain. The recombinant protein was expressed in inclusion bodies and purified as previously described (Boix, 2001), with some modifications (Palmer and Wingfield, 2004). Briefly, from an overnight culture, bacteria were grown in Terrific broth (TB), containing 400 μg/ml ampicillin at 37°C, and induced with 1 mM IPTG after reaching an OD_600nm_ around 0.6. After 4 h incubation, cells were harvested, and the pellet was incubated with 40 μg/ml of lysozyme in lysis buffer (10 mM Tris-HCl pH 8 and 2 mM EDTA) for 30 min. Then, cells were sonicated and centrifuged at 30.000× *g* for 30 min and the inclusion bodies were washed in 25 ml of lysis buffer with 1% Triton X-100 and 1 M urea for 30 min and collected by 30 min centrifugation at 22.000 g. This procedure was repeated until the supernatant was completely transparent, with a final washing step of 200 ml of lysis buffer without Triton X-100. Solubilization of the inclusion bodies was achieved by 2 h incubation of the pellet with 25 ml of Tris-acetate 100 mM, pH 8.5, 2 mM EDTA, 6 M guanidine hydrochloride and 80 mM of GSH at room temperature in a N_2_ atmosphere. The protein was then refolded for 72 h at 4°C by a rapid 100-fold dilution into 100 mM Tris/HCl, pH 8.5, 0.5 M of guanidinium chloride, and 0.5 M L-arginine, and oxidized glutathione (GSSG) was added to obtain a GSH/GSSG ratio of 4. The folded protein was then concentrated, buffer-exchanged against 150 mM sodium acetate, pH 5 and purified by cation-exchange chromatography on a Resource S (GE Healthcare) column equilibrated with the same buffer. The protein was eluted with a linear NaCl gradient from 0 to 2 M in 150 mM sodium acetate, pH 5. Protein purity was checked by SDS-PAGE gels and the protein fractions were desalted, lyophilized and stored at −20°C.

### 2.2 Crystallization trials

The three RNase 3/1 versions were resuspended in 20 mM cacodylate, pH 6.5 at 10 mg/ml for RNase 3/1-v1 and 7 mg/ml for RNases 3/1-v2 and -v3. The first trials were done at nanodrop scale (200 nL) using the sitting-drop method in 96-well plates following commercial high-throughput screening with an automated Crystal Phoenix (Art-Roberts Instruments, ARI) robot at the ALBA synchrotron facility and the Servei de Proteòmica i Biologia Estructural (IBB-UAB). Afterwards, the successful crystallization conditions from the screening plates were optimized by pH and precipitant concentration in 24-well plates using the hanging drop method. RNase 3/1-v2 and v3 needle-bar crystals grew after 5–20 days in similar precipitant conditions: 100 mM sodium citrate, pH 6.5, at different ammonium phosphate concentration of 1 and 1.5 M respectively. Another positive condition for RNase 3/1-v2, 100 mM sodium acetate, pH 5 and 1 M sodium chloride, gave column-bar crystal after few days. For RNase 3/1-v1, a big column crystal appeared in 100 mM sodium acetate, pH 4, and 1.5 M sodium chloride after several months at 18°C. The crystals were preserved in liquid nitrogen after cryoprotection by soaking into a mixture of glycerol 20% (v/v) and crystallization buffer.

### 2.3 Data collection, processing and protein structure determination

Crystal data were collected at the BL13-XALOC beam line station (ALBA synchrotron) at 100 K using a Pilatus 6M detector (Dectris®). The obtained data were processed using *XDS* (Kabsch, 2010).

For phase determination, molecular replacement method with Phaser-MR from the PHENIX software package (Adams et al., 2010) was applied using the following templates: RNase A structure (3DH5.pdb) for RNase 3/1-v1, and a model created by MODELLER (Webb and Sali, 2016) of both RNase 3/1-v2 and v3 between RNase 1 (2K11.pdb) (Kövér et al., 2008) and RNase 3 (1QMT.pdb) (Boix et al., 1999). The initial structures were improved by iterative cycles of automated refinement using *phenix.refine* from the PHENIX software package combined with manual building using Coot (Emsley and Cowtan, 2004), until the R-free could no longer be improved. Crystallographic statistics of data processing and molecular refinement are listed in Supplementary Table S1.

### 2.4 Molecular modelling

Molecular modelling was performed with HADDOCK web server version 2.2 (Van Zundert et al., 2016). The HADDOCK software (acronym of High Ambiguity-Driven protein–protein DOCKing) calculates docking interfaces for protein–nucleic acid complexes based on experimental knowledge in the form of ambiguous interaction restraints. Furthermore, HADDOCK uses multiple stages of docking, in which an initial rigid body docking is followed by a semiflexible refinement docking stage. Alternatively, blind protein-protein rigid body docking was performed using *ClusPro* 2.0 (Desta et al., 2020). Last, binding energy of predicted protein-protein complexes was estimated with PRODIGY (Xue et al., 2016).

To analyze the RNase 3/1 variants in complex with the RNHI inhibitor, both HADDOCK and *ClusPro* programs were applied. The inhibitor coordinates were taken from the RNase 2-inhibitor complex (2BEX.pdb) (Iyer et al., 2005), sharing RNase 2 and RNase 3 a 67% amino acid identity. Results were compared with the RNase 1-RNHI complex (2Q4G.pdb) (Johnson et al., 2007).

To investigate the structural differences between the three RNase 3/1 variants that account for their distinct catalytic activities on dinucleotides (Prats-Ejarque et al., 2021), we used HADDOCK and PRODIGY. First, CpA and UpA were obtained from crystallographic data, CpA from 1RPG.pdb (Zegers et al., 1994) and UpA from 11BA.pdb (Vitagliano et al., 1998). Regarding CpG and UpG, both were created with elBOW (Moriarty et al., 2009). Dinucleotides were docked to the specific binding sites of the three RNase 3/1 versions using HADDOCK. The active amino acid residues were assigned in basis of the interactions observed in the RNase A –CpA crystal structure (1RPG.pdb) (Zegers et al., 1994), while passive amino acid residues were automatically defined by the program. Ambiguous interaction restraints (AIR) were generated on the basis of a list of selected active and passive residues for both protein and nucleic acids. Previously, the 3D structure of RNase 3/1-v2 was completed by addition of the last 2 residues that were missing in the solved crystallographic structure. The resulting complexes created were inspected using PRODIGY and *PyMol 1.7.2.*

## 3 Results

**FIGURE 1:**
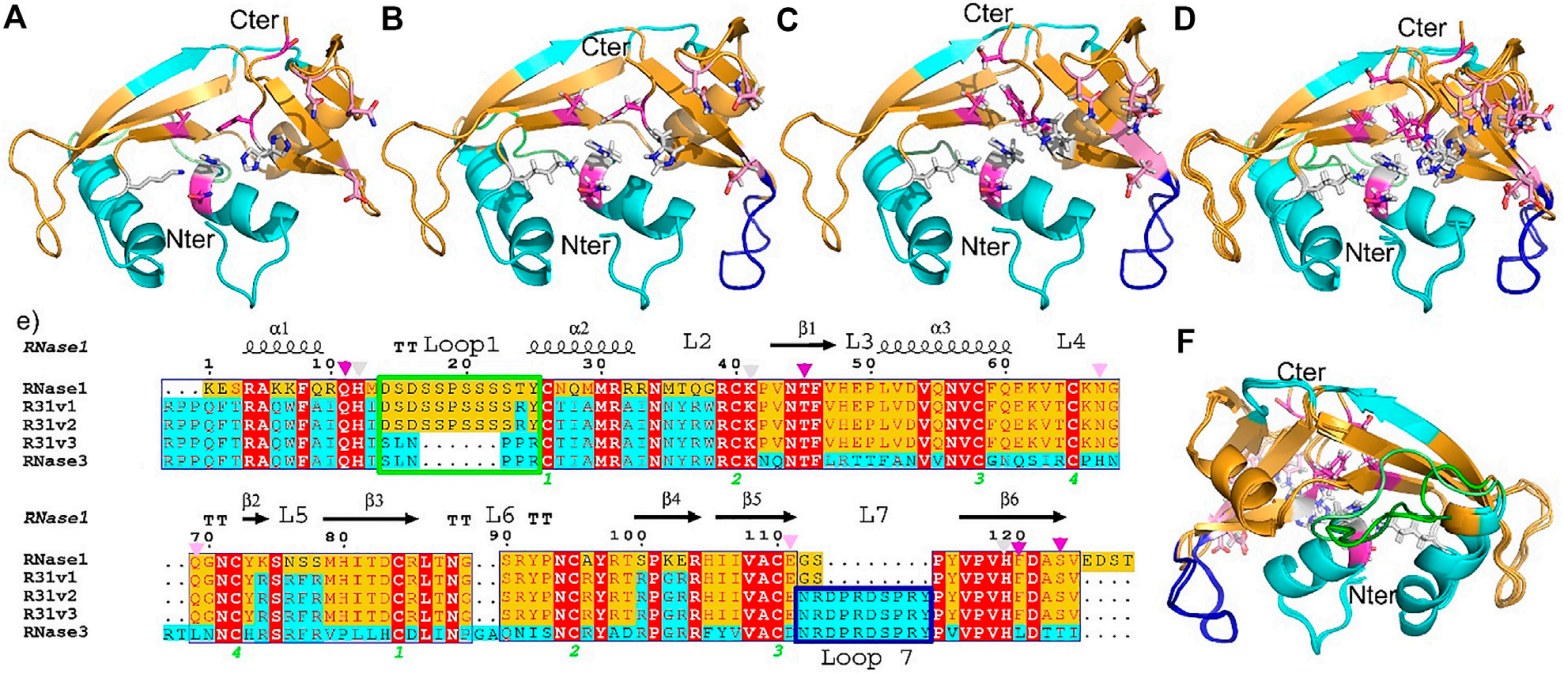
RNase 3/1 versions 1, 2 and 3 structures are showed in panel **(A–C)** respectively. Panels **(D,F)** show an image of all three RNases superposed, facing forwards and backwards respectively. Backbone region corresponding to RNase 1 and RNase 3 are indicated in gold and cyan respectively. RNase 1 Loop 1 (R1-L1) is boxed in green and RNase 3 Loop 7 (R3-L7) in dark blue. The color code is the same in both the structures and the sequences. Sequence alignment is shown in panel **(E)**. Catalytic residues are labelled with a triangle and the loops are marked with a square. The side chains of RNase A catalytic site are colored in light gray, B1 pocket residues in magenta, and B2 residues in pink. The sequences of the three versions are aligned with both parental RNases. Conserved residues are highlighted with a red box. The secondary structure of RNase 1 is plotted at the top of the alignment.

A first RNase hybrid construct was engineered to combine the high catalytic activity of human RNase 1 and the antimicrobial properties of RNase 3 (Prats-Ejarque et al., 2019a). Based on the RNase 1 scaffold we incorporated specific regions essential for RNase 3 unique anti-pathogen activities within the RNase A superfamily (Boix et al., 2012) (see Supplementary Table S2). Briefly, we incorporated the N-terminus of RNase 3 (1–45) that encompasses the main antimicrobial region of the protein and consists of the first two helices (Torrent et al., 2009, 2013; Sánchez et al., 2011), which include an aggregation prone and a lipopolysaccharide (LPS) binding sequence (Torrent et al., 2012; Pulido et al., 2016). On the other hand, the hybrid construct conserved the flexible loop L1, ranging from residues 17 to 26 of RNase 1 and reported essential for the protein catalytic activity (Doucet et al., 2009; Gagné et al., 2015). Complementarily, several key Arg residues identified to participate in RNase 3 interaction to microbial anionic surfaces (Carreras et al., 2003, 2005; Boix et al., 2012) were incorporated into the RNase 1 skeleton (see Figure 1 and Supplementary Table S2).

**FIGURE 2:**
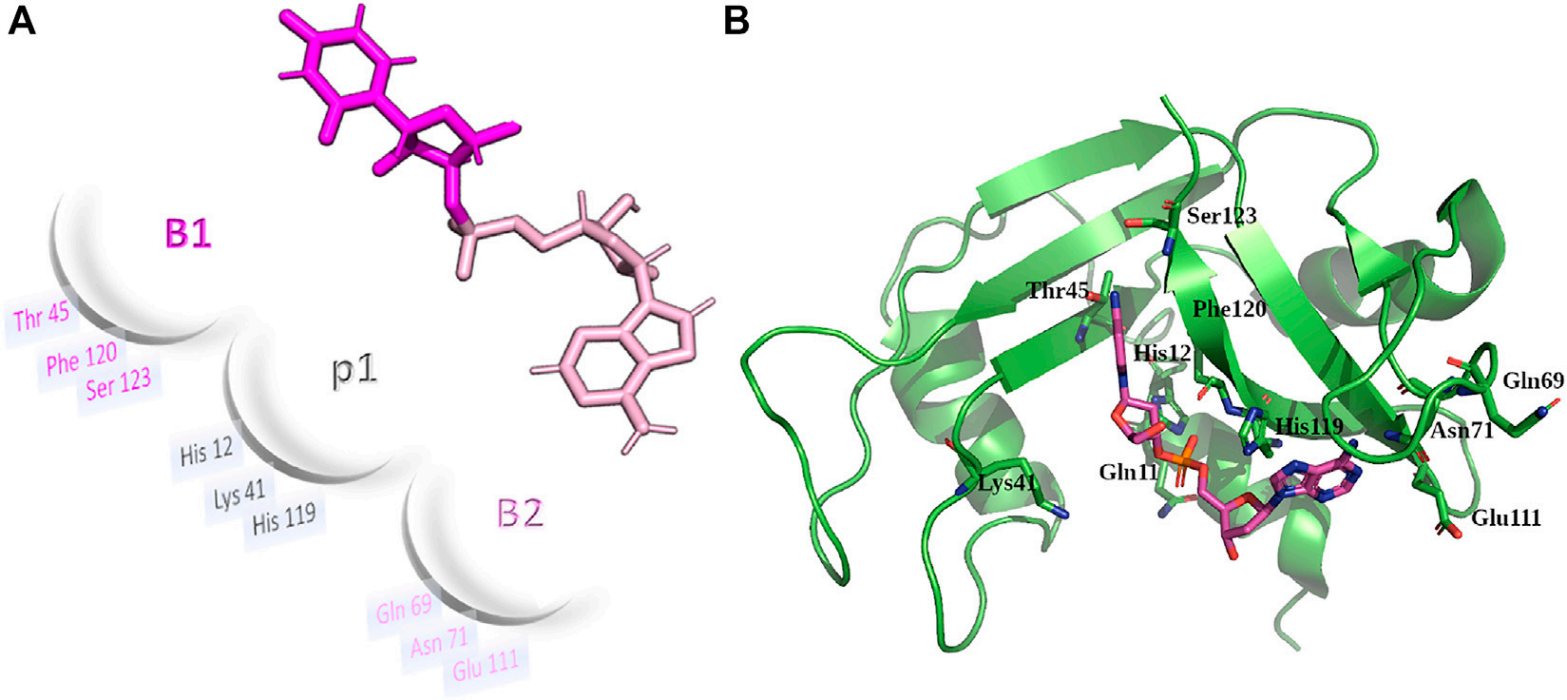
**(A)** Schematic illustration and 3D representation of RNase 1 subsite arrangement for main phosphate and base binding sites. UpA dinucleotide is depicted as a reference model, Colors for B1 and B2 sites (pink and magenta respectively) are taken as a reference. **(B)** RNase 1 structure (2K11.pdb) overlapped with CpA from RNase A-CpA complex (1RPG.pdb). Key residues from B1, p1 and B2 subsites are labelled.

Next, a second variant (RNase 3/1-v2) was designed to incorporate loop L7, a region characteristic of RNase 3 and shared with other antimicrobial RNases, but absent in RNase 1 (Carreras et al., 2003; Boix and Nogués, 2007). Last, a third version was designed, where the flexible loop L1 of RNase 1 was removed (Figure 1). All the three variants conserved the regions identified in RNase A to belong to the primary and secondary base binding pockets (Boix et al., 2013; Prats-Ejarque et al., 2019b), named as B1 and B2 sites respectively (see Figure 2).

### 3.1 Overall structure analysis of the three RNase 3/1 chimeras

The three RNase 3/1 variants were solved by X-ray crystallography (see Supplementary Table S1 for final processing and refinement statistics). RNase 3/1-v2 was solved in the free form and in the presence of phosphate anions. All crystals diffracted to atomic resolution (1.1–1.5 Å): RNase 3/1-v1: 6YMT, RNase 3/1-v2: 6YBE, RNase 3/1-v2 phosphate complex: 6YBC and RNase 3/1-v3: 6SSN. Interestingly, we observed a wide diversity in crystal morphology among the three chimeras (Supplementary Table S1). To note, RNase 3/1-v1 crystals had the biggest cell size, containing twelve chains in the asymmetric unit. The solved crystal structures confirmed that all the chimera proteins reproduce the kidney-shaped folding typical of the RNase A superfamily (Figures 1A–C and Supplementary Figure S1) and thus keep the fold of the templates used in their design, as suggested by circular dichroism and molecular dynamics analysis (Prats-Ejarque et al., 2021). Analysis of secondary structure elements and topological organization also highlighted that the chimeras mostly reproduced equivalent patterns to the parental proteins, with only slight differences at the protein N and C-terminus ends (Supplementary Figure S2).

Structural alignment was performed between all protein structures and between chains within each asymmetric unit using the software LSQMAN (Kleywegt, 1996) (Supplementary Figure S1). In addition, each RNase 3/1 version was compared respect to the template-based design structures: RNase 1 (2K11.pdb) and RNase 3 (1QMT.pdb). The structural variability was determined by comparing the RMSD of the α-carbon peptide chains (Supplementary Tables S3, S4). Overlapping of all the crystallography structures (Figures 1D,F and Supplementary Figure S3) indicated a good agreement, with RMSD between the respective backbones ranging from 0.7 to 1.1 Å.

#### 3.1.1 RNase 3/1-v1 structure

The first version of RNase 3/1-v1 has twelve peptide chains in the asymmetric unit. The maximum RMSD deviation among backbone chains was 0.7 (Supplementary Table S3), being the highest variation located within the last five C-terminal residues (Supplementary Figure S1A), suggesting that this region has a higher flexibility in solution. To note, the last beta strand was interrupted in residue Phe123, turning the C-terminal tail towards the active site cavity and, in particular, to the B2 region. We should also bear in mind that in the original RNase 1 structure the C-terminal beta strand includes two residues (Phe123 and Ser126) that are directly involved in the B1 cavity. Here, the location of these residues is displaced from their original position, suggesting an impact in the B1 base binding and selectivity, as later discussed. In addition, the C-terminal displacement might induce slight changes at the conformation of the L4 loop (66V-72Q), which conforms the B2 site, as later detailed. Surprisingly, the R1-L1 (D17-R27), predicted as highly flexible in RNase A studies (Doucet et al., 2009), is here perfectly superposed between the twelve chains (Supplementary Figure S1A).

#### 3.1.2 RNase 3/1-v2 structure

**FIGURE 3:**
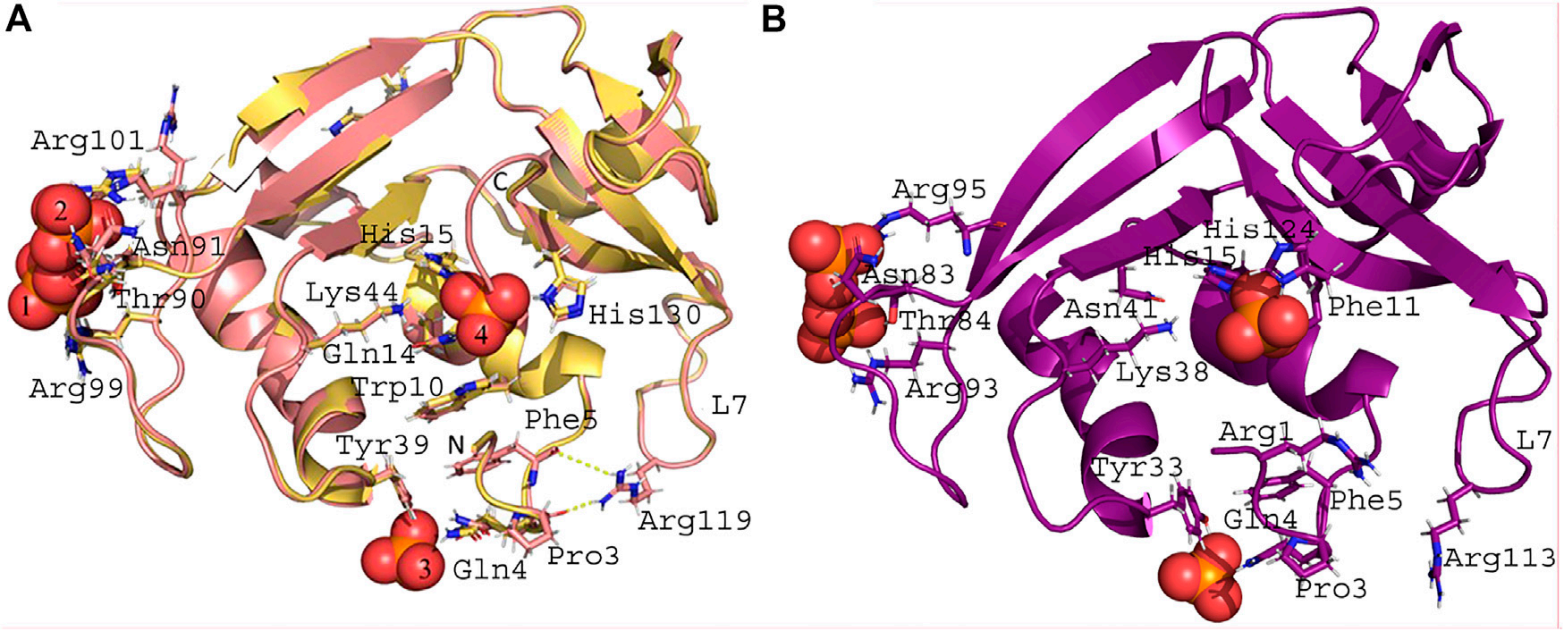
Crystal structures in complex with phosphate anions. **(A)** Crystal structure of RNase 3/1-v2. The golden colored structure corresponds to the RNase 3/1-v2 crystal obtained in the presence of phosphate, and the pale red structure corresponds to the one without phosphate. The PO_4_ n°1 is interacting with: Thr90, Asn91, and Arg99. The PO_4_ n°2 is interacting with Thr90, Asn91, and Arg101. The PO_4_ n°3 is interacting with: Gln4 and Tyr39. The PO_4_ n°4 is interacting with the catalytic triad (His15, Lys44, and His130), Trp10, and Gln14. Arg119 from the R3-L7 loop is interacting with the main chain of Pro3 and Phe5. All the listed residues have the same conformation in the two structures, except Arg101 that presents an alternative conformation in the structure without phosphate and interacts with PO_4_ n°2 in the complexed structure. **(B)** Crystal structures of RNase 3/1-v3. Phosphate n°1 interacts with Thr84 and Arg93, and PO_4_ n°4 with Arg95. Phosphate n°2 interacts with Gln4 and Tyr33. Phosphate n°3 interacts with the catalytic triad (His15, Lys38, and His124), Asn41, and Phe11. Arg113 (equivalent to Arg119 in -v2), within the R3-L7, cannot interact with Pro3 or Phe5.

Two crystal structures of RNase 3/1-v2 were solved, one free and the other in complex with phosphate ions (Figure 3A). Crystals from free and phosphate-bound structures were obtained in distinct crystallization conditions but belonged to the same space group (Supplementary Table S1). No significant differences at the structural level were observed, with an RMSD of 0.4 Å (Supplementary Figure S1B and Supplementary Table S4). In both cases the model is traced till residue Ala134, being shorter than its full sequence because no electron density is observed for the C-terminal end. Analysis by Mass Spectrometry confirmed the expected size of the protein; therefore, the absence of electron density is due to high mobility of the chain at this region. Therefore, the last β-strand is interrupted and Phe132 is displaced. As observed in RNase 3/1-v1, the residues Phe132 and Ser134 in -v2 variant have a different position than their RNase 1 counterparts, altering the B1 site cavity conformation. Interestingly, the R1-L1 (D17-R27) was hard to build in both crystal structures of RNase 3/1-v2 due to poor electron density map, in opposition to the equivalent L1 loop in RNase 3/1-v1. Regarding the R3-L7 (N115-F126) loop, this region adopts here a fixed position due to its interaction with the N-terminal. The H-bonds between Asp120 and Thr6 observed in both RNase 3 and the -v2 chimeras are reinforced here by a small hydrophobic patch created by Arg119 and Glu117 side chains around the methyl group of Thr6.

#### 3.1.3 RNase 3/1-v3 structure

Finally, RNase 3/1-v3 was crystallized in the presence of phosphate anions (Figure 3B). The structure has two molecules in the asymmetric unit with an RMSD between backbone chains of 0.27 Å. Again, this low value indicates no important variation due to crystal packing or distinct local flexibilities. Only the R3-L1 shows some small differences (Supplementary Figure S1C). The C terminal β-strand is conserved in this version, keeping the geometry of the B1 pocket. Besides, the R3-L7 loop has the same interactions with the N-terminal in both RNases 3/1-v2 and v3 (Figure 3). Indeed, the C_α_ backbone of both versions overlaps perfectly along all the loop (see Figure 1D).

Overall, apart from intrinsic differences due to the removal of the flexible R1-L1 loop but conservation of the R3-L7 loop, the peculiarities of -v3 chimera lie at the protein C-terminus. While in RNase 3/1-v3 there is an extended β-strand, in the first two chimeras the C-terminus end is disordered, determining significant changes at the B1 pocket.

#### 3.1.4 Analysis of phosphate interactions in RNases 3/1-v2 and v3

RNases 3/1-v2 and -v3 variants were obtained in the presence of phosphate anions (Figure 3). A close inspection of phosphate anions in RNase 3/1-v2 indicates that one phosphate anion is interacting with the catalytic triad (Figure 3A). The other phosphates are bound at the protein surface, where PO_4_ anions n° 1 and 2 might correspond to alternative positions of the same binding site. Comparison of free and phosphate bound RNase 3/1-v2 structures highlights Arg 101 residue, which gets fixed at only one position upon phosphate interaction. Interestingly, an equivalent distribution of PO_4_ anions is obtained in RNase 3/1-v3 (Figure 3B), where phosphate n° 3 is located at the active site. Presence of a phosphate anion interacting with the enzyme catalytic triad confirms the conservation of the active site groove in both -v2 and -v3 structures. The figure is also showing the specific interactions occurring between both protein N-terminus and the inserted R3-L7 loop, associated to RNase 3 antimicrobial activity.

### 3.2 Structural differences between the three versions of RNase 3/1 alter the substrate selectivity

**FIGURE 4:**
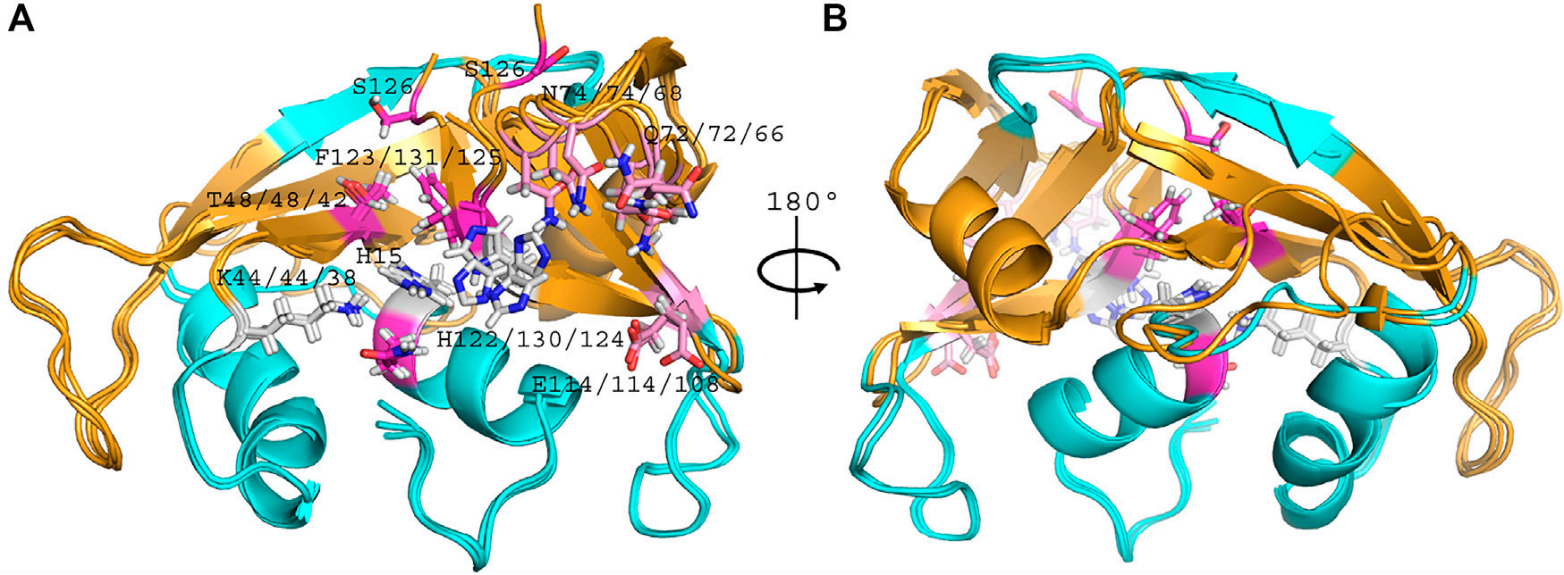
Overlapping of RNase 3/1-v1 (6YMT.pdb), RNase 3/1-v2 (6YBC.pdb) and RNase 3/1-v3 (6SSN.pdb) crystal structures. In light blue, the fragments of RNase 3, in orange, the RNase 1 skeleton. In grey, the catalytic triad, in pink the B1 subsite and in magenta the B2 subsite. The picture was drawn with *PyMOL 1.7.2* (Schrödinger, LLC).

Overall, side by side comparison of the three variants highlighted a good fit between their main chain backbone (Figure 4). Considering the non-significant variability between all chains of the asymmetric unit within each RNase 3/1 variant, all comparisons between the three chimeras were referred to the first chain of each structure.

A general overview by overlapping the three structures together with the parental RNase 1 and RNase 3 indicates that active site triad are at equivalent positions in all structures (see Table 1 for residue numbering equivalencies). Equivalent positions are also observed for B1, where the variants are mostly resembling RNase 1 rather than RNase 3 (all with an equivalent Thr to Thr45, whereas Phe120 in RNase 1 and variants are substituted by Leu129 in RNase 3). On the contrary, main and side chains are significantly different at B2 site for variants -v1 and -v2, whereas the last variant recovers the main chain conformation characteristic of B2 site, mimicking more closely RNase 3 structure in this region.

**TABLE 1:** Equivalencies for selected key residues and intramolecular interactions in all superimposed 3D structures.

RNase 1	RNase 3/1-v1	RNase 3/1-v2	RNase 3/1-v3	RNase 3
Ser3	Thr6	Thr6-Asp120	Thr6-Asp114	Thr6-Asp118
Arg4	Arg7-Glu114	Arg7-Glu114	Arg7-Glu108	Arg7-Asp112
Phe8	Phe11	Phe11	Phe11	Phe11
Gln11-Lys41	Gln14-Lys44	Gln14-Lys44	Gln14	Gln14-Lys38
His12	His15	His15	His15	His15
Tyr25	Tyr28	Tyr28	Pro21	Pro21
Lys41-Gln11	Lys44-Gln14	Lys44-Gln14	Lys38	Lys38-Gln14
Thr45-Asp83	Thr48-Asp86	Thr48-Asp86	Thr42-Asp80	Thr42-His82
His48	His51	His51	His45	Arg45
Lys66	Asn70	Asn70	Asn64	Asn65-Asp130
Gln69	Gln72	Gln72	Gln66	Asn69
Asn71	Gly73	Gly73	Asn68	Asn70
Tyr73-Tyr115	Tyr 76-Tyr118	Tyr76-Tyr126	Tyr70-Tyr120	His72
Asp83-Thr45	Asp86-Thr48/Arg107	Asp86-Thr48/Arg107	Asp80-Thr42/Arg101	His82-Thr42
Pro101	Pro104	Pro104	Pro98	Pro102
Lys102	Gly105	Gly105	Gly99	Gly103
Ser100	Arg103	Arg103	Arg97	Arg101-Asp84
Arg104	Arg107-Asp86	Arg107-Asp86	Arg101-Asp80	Arg105
Glu111	Glu114	Glu114	Glu108	Asp112
Tyr115	Tyr118	Tyr126	Tyr120	Val124
His 119	His 122	His 130	His124	His128
Phe120	Phe123	Phe131	Phe125	Leu-129
Asp121-His119	Asp124	Asp132	Asp126-His124	Asp130-His122
Ser123	-	-	Ser128	Thr132

#### 3.2.1 Structural analysis of chimera catalytic properties

Next, we decided to explore in more detail the differences among the three constructs to interpret their distinct catalytic properties. Our previous kinetic characterization using polyuridine (poly(U)) substrate indicated a gradual decrease of activity from RNase 3/1-v1 to -v3, where -v1 retained about a 75% of the catalytic activity from the parental RNase 1 (Prats-Ejarque et al., 2021). Interestingly, insertion of RNase 3 regions into RNase 1 scaffold not only diminished its catalytic efficiency but also altered the base specificity respect to the parental protein, as revealed by assessment of the protein activity against dinucleotides [see Table 2; (Prats-Ejarque et al., 2021)]. Here, based on the availability of the three crystal structures, we aimed to explain the observed shift in base specificity.

**TABLE 2:** U/C and A/G ratios were calculated from the kinetic results of (Prats-Ejarque et al., 2021) by comparison of the cleavage of UpA vs. CpA to analyse the U/C preference at B1 and the cleavage of UpA vs. UpG for analysis of A/G preference at B2.

Catalytic activity ratios at the B1 and B2 sites					
	RNase 1	RNase 3	RNase 3/1-v1	RNase 3/1-v2	RNase 3/1-v3
U/C	10.02	3.13	6.83	13.47	25.27
A/G	1322.34	∞	207.37	757.68	353.18

Previous kinetic data using CpA, UpA and UpG dinucleotides indicated for all the chimeras an overall preference for uridine vs. cytidine at B1 site and a preference for adenine vs. guanine at B2 site (Table 2). In particular, an increase in uridine preference was observed from -v1 to -v3, where the latest version had even a more pronounced U > C ratio than the parental proteins. On its turn, the A>G discrimination among variants showed less dispersion and was in all cases lower than the parental proteins [Table 2, (Prats-Ejarque et al., 2021)]. Overall, and surprisingly, the addition of the R3-L7 (-v2 and -v3) shows a more pronounced reduction of the catalytic activity than the deletion of the flexible R1-L1 loop (-v3), reported as important for the catalytic activity of the protein (Gagné and Doucet, 2013). However, the absence of the R3-L7 loop may indirectly alter the N-terminus position, taking into account the interactions between the C and N-terminus of RNase 3 observed by both NMR and X-ray crystallography (Boix et al., 1999; García-Mayoral et al., 2010).

**FIGURE 5:**
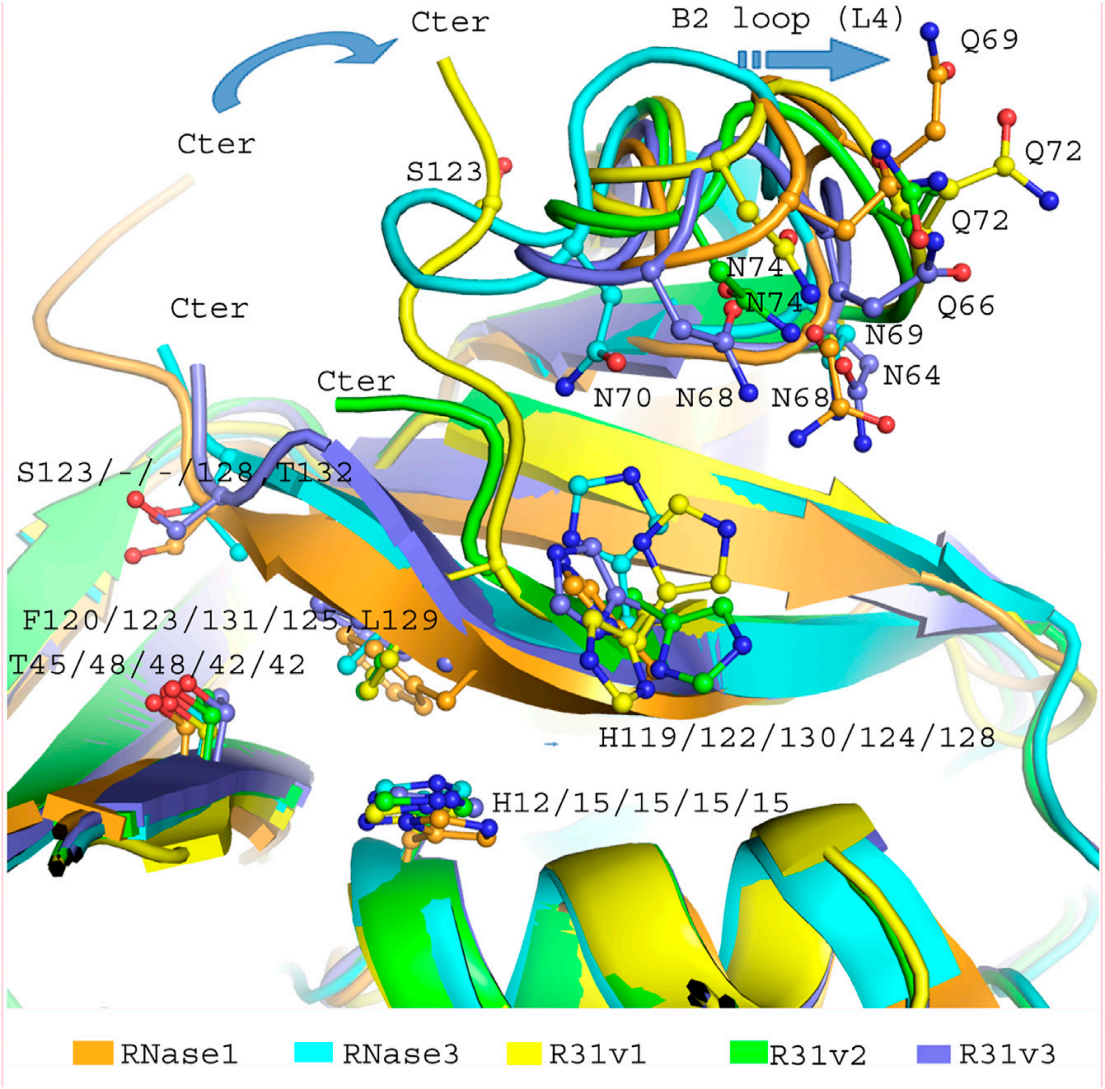
Overlapping of the B2 region of RNase 3/1-v1 (6YMT.pdb), RNase 3/1-v2 (6YBC.pdb) and RNase 3/1-v3 (6SSN.pdb) crystal structures. In light blue, the segments of RNase 3, in orange, the RNase 1 skeleton. Shared key residues for B1 and B2 interactions are indicated. The picture was drawn with PyMOL 1.7.2 (Schrödinger, LLC).

Figure 5 illustrates the distinct length in the C-terminus end within the 3 variants and how the shift in its position can alter the conformation of the loop associated to B2 binding site. In basis of the structural changes observed, the L7 loop would interact with the N-terminus, triggering a displacement of the first helix. In the case of RNase 3/1-v2, the α1 displacement would alter the active site and nearby residues position and might induce a decrease in activity. In the case of RNase 3/1-v3, the replacement of the flexible loop of RNase 1 by the shorter and more rigid loop of RNase 3 reduces the capacity of the first two helixes to readapt the active site configuration, provoking a major displacement of both helixes and forming a hydrophobic core similar to RNase 3 one (Mallorquí-Fernández et al., 2000).

**FIGURE 6:**
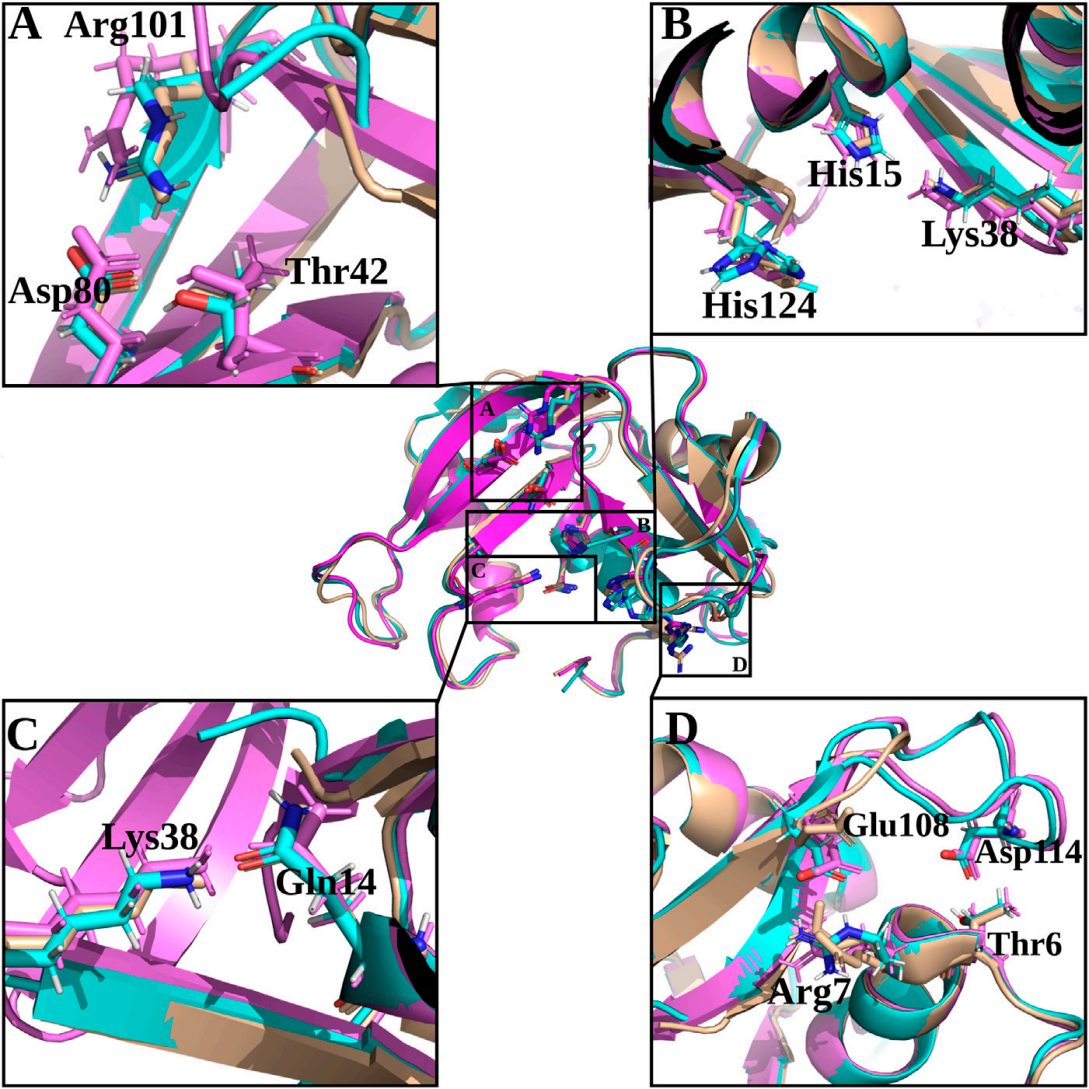
Structural details of selected key regions of RNases 3/1-v1, -v2 and -v3. RNase 3/1-v1 is shown in beige, RNase 3/1-v2 is shown in light blue and RNase 3/1-v3 is shown in magenta. Residues corresponding to -v3 are labelled. **(A)** Shows equivalent interactions between Arg-Asp in -v1 and v-2 but absent in -v3 that determine Thr orientation at B1 site; **(B)** shows the catalytic triad residues; **(C)** shows the Gln14-Lys38 interactions key for Lys38 position and intermediate stabilization during catalysis and **(D)** includes interactions between the N- and C-terminal regions that contribute to fix the L7 position present in RNases 3/1-v2 and -v3. The picture was drawn with *PyMOL 1.7.2* (Schrödinger, LLC).

On the other hand, despite all the variants conserved the catalytic triad configuration, together with a neighbouring Gln that interacts with the catalytic Lys (counterparts of Gln11 and Lys41 in RNase 1, see Table 1 and Figure 6, we do observe significant structural changes at the B1 binding pocket that might explain the significant increase of uridine preference at B_1_ subsite in both RNase 3/1-v2 and RNase 3/1-v3. Changes at the B1 site also arises due to the shift of the protein C-terminus location Although the first version shows an extended C-terminus, its position is displaced in relation to RNase 1. On its turn, -v2 has a shorter C-terminus end but cannot reproduce properly the parental protein conformation, whereas the last version provides a B1 environment more similar to the parental templates.

A closer look into the B1 environment shows how the specific orientation of Arg residues within the RNase 3 cationic patch inserted into the RNase 1 scaffold (see region 103–107 at Supplementary Table S2) can determine distinct electrostatic interactions that might contribute to the higher preference for uridine in all the chimeras. In the three variants we find an Arg-Asp interaction (Arg101/107- Asp80/86; see Table 1) that fixes the Asp side chain, which in its turn binds to Thr42/Thr48 (Figure 6 and Table 1). In contrast, in RNase 1, the Asp counterpart (Asp83) is directly hydrogen-bonded to Thr45, with no proximity of a cationic side chain. Indeed, in the position occupied by Arg101/Arg107 in the RNase 3/1 variants we find an anionic residue in RNase 1 (Ser123). Therefore, the chimera variants would provide a distinct environment for the pyrimidine base binding. Importantly, the Thr45 residue in RNase A is the main determinant for pyrimidine preference at B1 and Thr45/Asp83 alternative interactions would account for uridine/cytidine discrimination (Raines, 1998).

Although we successfully achieved in our initial purpose to design a RNase 3/1 chimera with enhanced catalytic activity and antimicrobial properties, the structural analysis revealed that RNase 3/1-v3 still retained some structural features similar to the parental RNase 3 that might limit its catalytic activity. A close inspection of the specific electrostatic interactions that might reduce the protein motion and flexibility at the active site revealed some key differences in comparison to the highly catalytically efficient RNase 1 counterpart. Apart from the structural differences commented above, we also observe for example how the region conformed by residues Lys102 and Arg 104 in RNase 1 corresponds to Gly103 and Arg105 in RNase 3 (Table 1). Equivalent residues are found in RNase 3/1-v3 (Gly99 and Arg101). Interestingly, we observe how electrostatic interactions between Arg101-Asp80 in RNase 3/1-v3 are fixing the Arg position at the binding site. Equivalent interactions are also visualized for the other variants. On the contrary, in RNase 1, the corresponding Arg is free and could directly interact with the substrate. We observe here how the position of the Arg might be influenced by the presence of a neighboring Lys in RNase 1, which is substituted in RNase 3 and all the versions by a Gly. Another interaction that might reduce the protein flexibility of RNase 3/1-v3 is Arg7-Glu108 pair that substitutes Arg7-Asp112 counterpart in RNase 3 (see Figure 6 and Table 1).

In addition, there is another interaction between the N- and C-terminus that may reduce the catalytic activity of the last two chimera versions: Thr6-Asp114 (that corresponds to Thr6-Asp118 in RNase 3). This interaction is fixing the R3-L7 loop with the N-terminus in the last two chimeras (Figure 6) and might considerably reduce the protein flexibility and potential substrate interaction at the active site. The L7 loop is absent in RNase 1 and therefore in this protein the N-terminus would have more freedom to move.

Another key residue associated to active site flexibility and catalytic efficiency is His48 in RNase 1 (Gagné and Doucet, 2013). This residue conforms a hinge region that facilitates the opening and closing of the active site groove upon protonation-deprotonation. The residue is conserved in all the three versions (His51 in -v1/-v2 and His45 in -v3) but is substituted by an Arg in RNase 3 (Table 1). However, His45 hinge role cannot be fulfilled in -v3 due to the absence of the RNase 1-L1 segment. This might partly contribute to the significantly lower catalytic activity of both RNase 3 and -v3 chimera.

#### 3.2.2 Comparative analysis of main (B1) and secondary (B2) base subsites by molecular docking

Next, a structural analysis by molecular docking was performed to better analyze the observed differences among the chimera catalytic efficiencies (Table 2). HADDOCK server was used to determine *in silico* the interaction of the three RNase 3/1 structures with dinucleotides (CpA, UpA, CpG and UpG) (Supplementary Table S5). Predicted interaction energies highlighted a similar affinity for all the tested dinucleotides. In relation to the base at the B1 site, results from the predicted protein-dinucleotide complexes revealed a slightly higher preference for uridine over cytidine for the three versions of RNase 3/1, supporting the calculated experimental catalytic activity ratio (Table 2). In particular, comparison of the estimated energies for electrostatic interactions by HADDOCK using CpA and UpA indicated small but significant differences for RNase 3/1-v2 and RNase 3/1-v3 (Supplementary Table S5). Respect to the B2 site, the estimated electrostatic energies indicated a higher affinity when adenine is at the B2 site for all the chimeras (see CpA vs. CpG and UpA vs. UpG in Supplementary Table S5). Remarkably, RNase 3/1-v2 is the version which shows higher differences in the calculated electrostatic interaction energies (-124.3 for UpA vs. -78.1 for UpG), in accordance with the preference for adenine versus guanine in the kinetic assays (Table 2). However, the overall differences in the estimated binding energies are quite small and do not serve to fully interpret the experimental kinetic results. Undoubtedly, the impaired catalytic efficiency of RNases 3/1 versions when a guanine base is located at the B2 subsite would not only depend on a lower interaction energy with the substrate, but also on the position of the catalytic triad respect to the phosphodiester bond.

**FIGURE 7:**
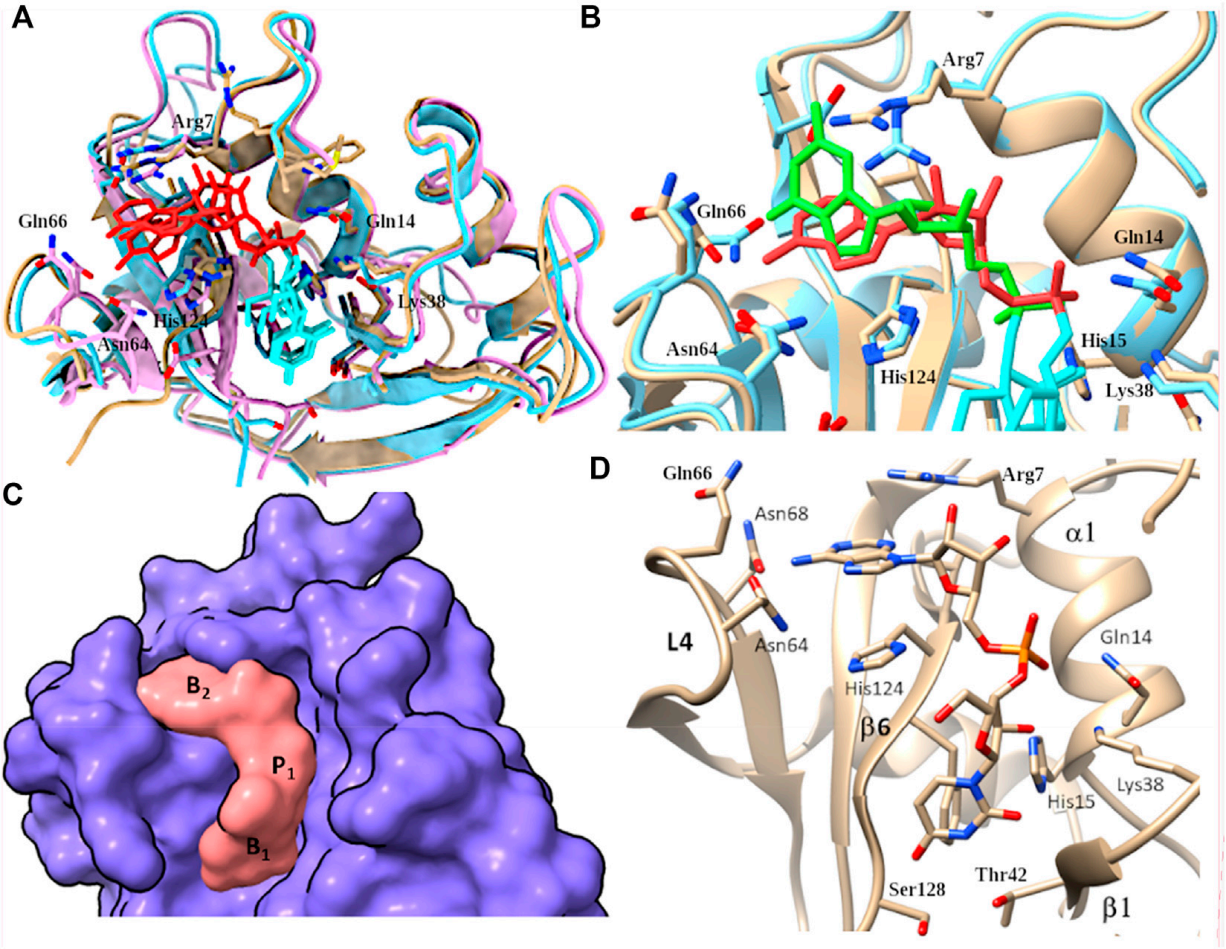
**(A)** Overlapping of predicted complexes with UpA and RNase 3/1-v1, -v2 and -v3 variants colored in beige, light blue and magenta respectively. Dinucleotide UpA is shown in light blue (Uridine) and red (adenine). **(B)** Overlap of RNase 3/1-v3 complex with UpA and UpG respectively. RNase 3/1-v3 is colored in beige in UpA and in blue in UpG complex. UpA is colored in orange and UpG is colored in green. **(C)** Surface representation to illustrate the complementarity between RNase 3/1-v3 surface and the dinucleotide UpA. Location of B1, B2 and p1 sites is indicated. **(D)** detail of predicted RNase 3/1-v3 in complex with UpA.

Last, docking results were analyzed to identify the residues involved in base binding. Taking the predicted UpA complexes as a reference, we can see how the dinucleotide is nicely located within the groove conformed by L4 loop and β6 for the three chimera complexes (Figure 7).

Overall, by comparison of all the predicted complexes we can conclude that the base location at B1 site is nicely overlapping for all structures, while there is a higher variability at the B2 site (Figures 7A,B). In addition, side-by-side comparison of interacting residues using *LigPlot* ^*+*^ (Laskowski and Swindells, 2011) highlighted the main shared contributing regions (see Supplementary Figure S4). In each RNase 3/1 chimera we can visualize the contribution of the Thr (Thr 45 in RNase 1) and potential interactions at the main chain of previous residue. Complementarily, in B1 pocket we observe the contribution of stacking interactions with Phe120 in RNase 1 and equivalent counterparts in the three variants (Phe123/Phe131 and Phe125 respectively). Overall, predictions corroborated that the last chimera is the one that better mimics the B1 cavity (Figure 7).

Regarding the interactions with the purine base at B2 site, we observe how, even if the main residues identified in RNase A for adenine binding (Gln69, Asn71 and Glu111) are conserved (Figures 1, 7D), differences in the neighboring residues can alter the final interaction mode (Supplementary Figure S4). In particular, significant changes at the main residue that provides selectivity for purine binding (Asn71 in RNase 1) are observed in the first two chimeras. In fact, superimposition of the three structures indicates that only Asn68 in the last version might provide equivalent interactions to Asn71 to bind to the purine base (see Table 1). Besides, counterparts of Gln69 in the chimeras are not oriented towards the base. In contrast, Glu111 in RNase 1 is conserved in all the three versions. Nevertheless, in the first version the side chain is too distant to the base to provide equivalent interactions. This might be due to the presence of Arg7 and its counterparts in all the chimeras, which can interact with the Glu residue. Finally, in B2 pocket we find the contribution of His119 that performs stacking interactions with the adenine, an interaction mimicked by all the variants (Figure 7). Nonetheless, comparison of the predicted complexes for the three variants with UpA highlights that the last version is the one that provide a higher number of interacting residues (Supplementary Figure S5).

Last, predictions of UpA and UpG complexes indicated how the interactions with the guanine base at B2 can alter the relative orientation of the purine base and impede proper base stacking interactions with the catalytic His. In addition, in the UpG complex we observe the displacement of phosphate location, which might prevent optimum interactions with catalytic triad residues (Figure 7B). However, molecular modelling did not serve to fully understand previous kinetic results that indicated a reduction in the chimera discrimination between adenine and guanine. As commented before, this might be due to the presence of a close Arg (Arg 7 in all the variants) that could interact with RNase 1-Glu111 counterpart (Glu114/Glu108 in the variants) and shift its original position.

### 3.3 The three RNase 3/1 chimera retained the overall interaction with the RNHI inhibitor

Last, the chimeras were analyzed to compare their interaction mode with the human RNase inhibitor (RNHI). RNHI is a proteinaceous inhibitor ubiquitous in the cytosol of somatic cells that binds in a 1:1 stoichiometry and protects cells from potential toxicity of secretory RNases (Lomax et al., 2014; Thomas et al., 2016).

Molecular docking was performed using both HADDOCK and *ClusPro* software. First, complexes were predicted using HADDOCK. Free RNHI crystal structure (1Z7X.pdb) was selected. Active and passive residues at the protein interface were predicted using the CPORT program [consensus prediction of Interface Residues (de Vries and Bonvin, 2011)]. The docking was performed using the Prediction interface by HADDOCK, based on the residues selected by CPORT. The predicted interactions between the inhibitor and the RNases were analyzed using the Arpeggio webserver (Jubb et al., 2017) and the COCOMAPS webserver (Vangone et al., 2011) selecting a 6 Å threshold.

All predicted complexes revealed a similar buried area, ranging from 3,600 to 4,000 Ǻ^2^. As reported for previously solved crystal complexes of RNase A family members with RNHI, the main region for interaction relies on the L6 loop, which encompasses residues 86–99 in RNase 1. Secondarily, we observed interactions within the L2 loop, corresponding to residues 34 to 41 in RNase 1, together with few additional residues at the N and C-terminus of the protein (Kobe and Deisenhofer, 1996; Papageorgiou et al., 1997; Iyer et al., 2005).

**FIGURE 8:**
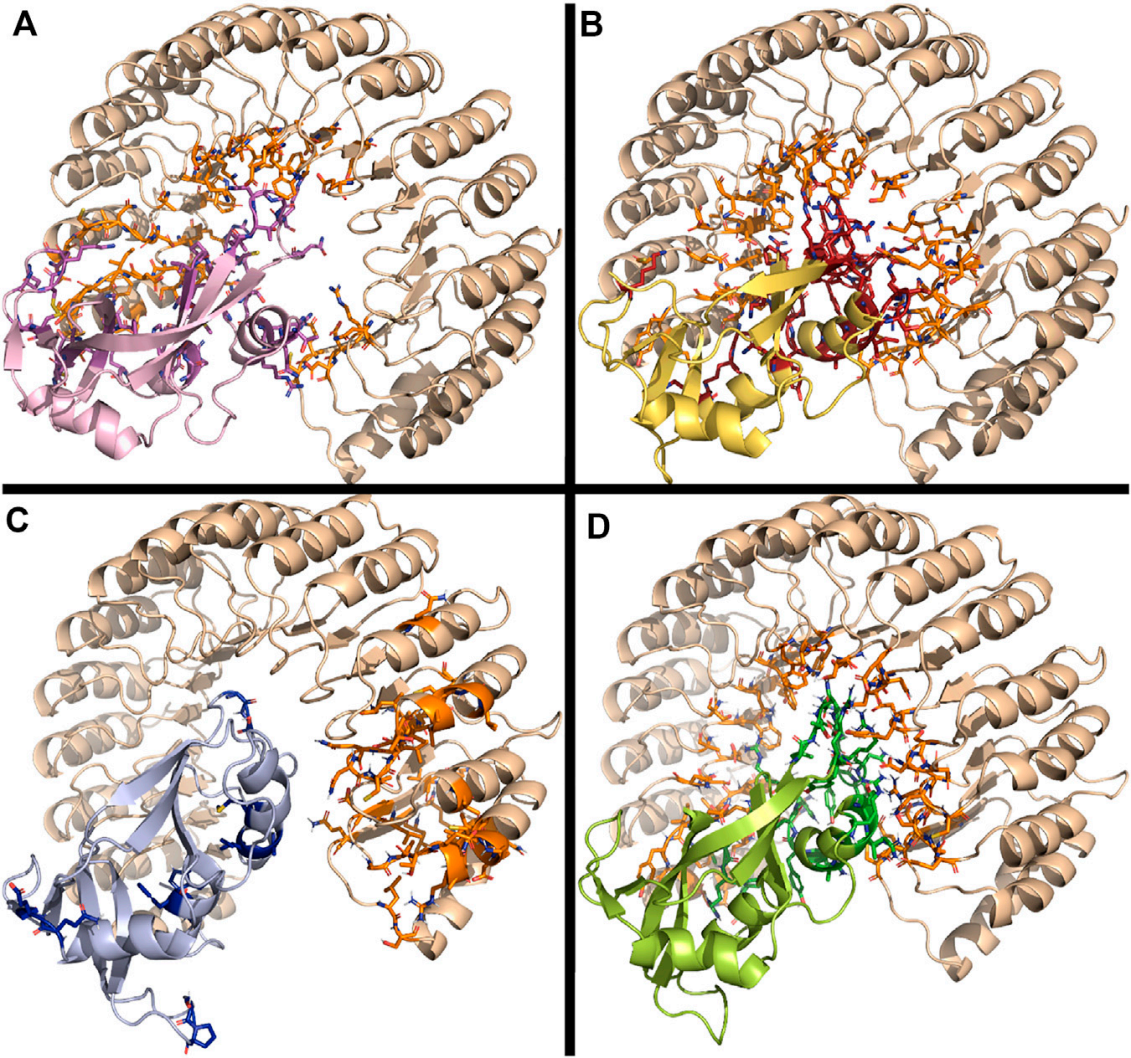
Predicted interaction complex of RNases with the RNHI using *ClusPro*. **(A)** RNase 1, **(B)** RNase 3/1-v1 **(C)** RNase 3/1-v2 and **(D)** RNase 3/1-v3. Interacting side chains are depicted. Figure was drawn with *PyMOL 1.7.2* (Schrödinger, LLC).

Rigid blind docking using *ClusPro* corroborated the predicted favoured orientations for all the complexes predicted by HADDOCK (Figure 8). Following, PRODIGY was applied to analyze the RNase-inhibitor interfaces (Supplementary Table S6), which were further inspected using *LigPlot* ^*+*^ (Laskowski and Swindells, 2011). Predicted interacting residues were compared with the solved crystal structure of human RNase 1 in complex with the RNH1 (2Q4G.pdb) (Johnson et al., 2007). As no solved 3D structure of RNase 3-RNHI is currently available, we used RNase 2-RNHI crystal structure (2BEX.pdb) to predict RNase 3 potential interaction mode, taking into consideration that both RNases 2 and 3 proteins share a 70% amino acid identity. The predicted RNase 3 complex showed a good agreement with RNase 2-RNHI solved structure (see Supplementary Figures S5, S6 and Annex 1).

Overall, side-by-side comparison of the modelled interaction mode for the three RNase 3/1 variants highlighted the residue equivalencies at the protein-protein interfaces (Annex 1). Remarkably, all the key regions for RNHI interaction in RNase 1 complex were retained in the RNase 3/1 chimeras (Figure 8), where loops 2 and 6 are standing out, whereas the R1-L1 and R3-L7 loops were not involved in the inhibitor binding.

Interestingly, blind docking predicted an equivalent protein orientation for all the complexes and shared interacting regions. Particular attention should be drawn at Tyr33 and Trp35 within the RNase 3 structure. Interestingly, the loop 2 region (32–40) in RNase 3 is also predicted to participate in RNHI binding, with interactions at Asn32, Arg36, Asn39 and Gln40 (Supplementary Figure S6). In addition, the other key loop for inhibitor binding reported for all studied RNases (L6) is mostly conserved between RNases 2 and 3, but significantly differs from RNase 1 and RNase 3. This is mostly due to a two residues insertion unique for both RNases among the family members. To note, all RNase 3/1 constructs reproduce RNase 1 sequence at L6 region but take the L2 region from RNase 3. Nonetheless, all structures show a similar predicted binding energy to RNHI (Supplementary Table S6).

## 4 Discussion

Antimicrobial proteins usually stand out for their pleiotropic properties, a trait that can be exploited to target antimicrobial resistance (Li et al., 2020). Within this context, RNase A superfamily members that combine enzymatic and antimicrobial properties can be engineered to develop alternative antibiotics (Prats-Ejarque et al., 2019a). A wealth of information from previous structure-functional and comparative evolution studies has enabled us to identify the main regions ascribed to antimicrobial action within the RNase A superfamily. Our previous work on RNase 3 by site directed mutagenesis, limited proteolysis and peptide design has facilitated the mapping of the functional regions (Sánchez et al., 2011; Torrent et al., 2011, 2013).

Based on this previous knowledge, our research group has designed the first RNase chimera that combines the enzymatic activity of RNase 1 with the antimicrobial properties of RNase 3 (Prats-Ejarque et al., 2019a). Two additional variants were later designed, where variant 3 combined the best of both parental proteins, achieving similar antimicrobial properties to RNase 3 (Prats-Ejarque et al., 2021).

Interestingly, the present structural analysis confirms that the incorporation of selected RNase 3 regions into the RNase 1 skeleton preserves the configuration of the active site centre (Figure 4). Besides, we obtained two crystal structures in the presence of phosphate anions and confirmed the formation of proper side chain interactions at the catalytic p1 site (Figure 3). However, whereas equivalent residues are positioned to conform the enzyme catalytic triad in the three chimeras (Figure 6), we observe significant deviations at the main and secondary base binding sites (Table 1). In particular, a significant change at the secondary base B2 subsite is observed, which might be driven by a shift of the protein C-terminus position (Figure 5). This is mostly remarkable for the first two variants, whereas variant 3 mostly recovers the main chain conformation characteristic of the B2 site, closely mimicking the parental RNase 3. In particular, a higher mobility and reduced stability was predicted for RNase 3/1-v2 by previous molecular dynamics and circular dichroism assays (Prats-Ejarque et al., 2021), which could be attributed to the insertion of R3-L7 loop while maintaining the R1-L1 loop. In contrast, RNase 3/1-v3, thanks to the absence of the extended and more flexible L1 loop of RNase 1, might have a reduced protein motion and hence a reduced catalytic efficiency. Although the hinge residue His48 identified in the RNase A scaffold to mediate the protein motion (Gagné et al., 2012) is present in the three variants, this residue in the latest variant cannot perform equivalent interactions due to the absence of R1-L1. Therefore, the comparative structural analysis of the three RNase 3/1 constructs corroborates the key role of the RNase 1 L1 loop for the protein motion and active site flexibility, which may facilitate the substrate binding and product release (Doucet et al., 2009; Gagné et al., 2012; Gagné and Doucet, 2013). On the other hand, the N-terminus in all the variants perfectly reproduces the conformation characteristic of RNase 3, while the protein C-terminus preserves most of RNase 1 features. Here it is worth mentioning the correlation reported between the sequence identity within the last residues of the protein in several family members and the enzyme catalytic efficiency (Allen et al., 1994). Previous structural and molecular dynamics studies have illustrated how the last protein beta strand (β6) is shaping both the B1 and B2 cavities. Although the residue Asp121 is conserved in all family members (Table 1) and is considered essential for interactions with the catalytic His119, the side chain conformation can induce significant changes on the subsite environment. A close inspection of this region in the three variants shows how the length of the β6 can determine a shift in the Asp side chain orientation (Figure 5). In fact, only in the third variant the Asp side chain provides proper interactions with the second catalytic His. The specific location of the C-terminus extension was associated to active site blockage and drastic decrease of catalytic activity in RNase 5, another family member, also called Angiogenin due to its angiogenic properties (Garnett and Raines, 2021). Interestingly, Acharya and co-workers demonstrated how substitution of the C-terminus end in RNase 5 by the RNase 2 counterpart was able to remove the active site blockage (Thiyagarajan and Acharya, 2013). Removal of the last four amino acids of RNase 1 also enhanced its catalytic activity (Bal and Batra, 1997). Here, the comparative structural analysis of the three RNase 3/1 variants demonstrates how specific interactions at the B1 site can determine the B2 site groove configuration, at the interface between the β6 and the L4 regions. Interestingly, while the B1 pocket is more clearly defined, we observe a higher variability at the predicted dinucleotide position at the B2 site for all the variants (Figure 7A). On the other hand, the molecular modelling analysis helped us to interpret the previous kinetic results, indicating that adenine versus guanine discrimination is partly influenced by the phosphate location at the p1 site and the relative orientation of the second catalytic His respect to the purine ring. Besides, we observe how proper interactions with the adenine base at the B2 site are also driven by L4 exposed residues (Figure 7D).

Contribution of L4 loop in B2 site was also demonstrated by Acharya and co-workers by the design of an RNase5/RNase A chimera. In that work the researchers substituted the angiogenic domain of RNase 5 with the RNase A secondary base region to introduce a purine binding site (Holloway et al., 2002). The chimera lost its angiogenin property but enhanced its catalytic activity. In this line, we find in the literature other successful chimera within the family to enhance enzymatic activity; for example, by replacing the N-terminus of Onconase, a frog RNase with antitumoral properties, by its RNase 1 counterpart (Boix et al., 1996; Esposito et al., 2019). Selective engineering at the N- and C-terminus ends has also been exploited to obtain artificial dimers by domain swapping (Merlino et al., 2012; Gotte and Menegazzi, 2019). Other RNase conjugates were also designed for cancer therapy, where the proteins were linked to a carrier for intracellular translocation (Rybak and Newton, 1999; Suzuki et al., 1999), receptor recognition (Forouharmehr et al., 2020), nuclear targeting (Vert et al., 2012) or cytosolic RNHI evasion (Targeted EVade^TM^ RNases). All these strategies generated RNase-based compounds with directed cytotoxicity towards malign cells (Castro et al., 2013; Montioli et al., 2021).

In contrast, in the present work we selected the structural elements linked to antimicrobial activity, while ensuring the protein non-toxicity to host cells. As commented above, RNase sequestration by the RNHI inhibitor protects cytosolic RNA from degradation (Rutkoski and Raines, 2008). This feature is essential to ensure the RNase non-toxicity to host cells. Here, we confirmed by structural analysis that the engineered variants retained the interaction with the inhibitor, as revealed by kinetic analysis (Prats-Ejarque et al., 2021). Previous structural studies of RNase-RNHI complexes demonstrated that the inhibitor leucine-reach repeat structure adopted an equivalent conformation and binding mode to diverse family members (Kobe and Deisenhofer, 1996; Papageorgiou et al., 1997; Iyer et al., 2005). Interestingly, all the engineered RNase 3/1 proteins showed a good fitting to the inhibitor cavity, as illustrated by the predicted complexes (Figure 8), where loops 2 and 6 stand out as shared conserved regions. Particular attention is drawn by region 32–40 at loop L2, which is conserved in all chimeras and belong to the identified LPS binding tag involved in the protein antimicrobial action (Pulido et al., 2016).

Thus, our results demonstrate that enzymatic and antimicrobial properties can be easily combined within a single molecule to obtain a functional novel antimicrobial compound. Overall, the combination of complementary antimicrobial activities can not only be used to target antimicrobial resistance but also provide a synergy action with other antibiotics already in the market (Prats-Ejarque et al., 2019a; Eller and Raines, 2020; Li et al., 2020).

## 5 Conclusion

Three RNase 3/1 chimera were successfully engineered to combine high catalytic and antimicrobial activities. Structure solving at atomic resolution reveals the conservation of the RNase A scaffold and the overall requisites for binding to the RNHI inhibitor. Comparative analysis of free and phosphate-bound complexes, together with molecular modeling using dinucleotides, confirm the active site architecture at the p1 site. Nonetheless, significant changes at the protein C-terminus position within the variants highlight its role in shaping both B1 and B2 substrate binding site specificity.

## Data Availability

The datasets presented in this study can be found in online repositories. The names of the repository/repositories and accession number(s) can be found below: http://www.wwpdb.org/, 6YMT http://www.wwpdb.org/, 6YBE http://www.wwpdb.org/, 6YBC http://www.wwpdb.org/, 6SSN.
