# Supplementary material for: Exploring the RNase A scaffold to combine catalytic and antimicrobial activities. Structural characterization of RNase 3/1 chimeras
Source: Front Mol Biosci. 2022 Sep 14;9:964717. doi: 10.3389/fmolb.2022.964717 (PMC9515509; doi:10.3389/fmolb.2022.964717)
Supplement: Supplementary file 2 [file DataSheet1.docx]

Supplementary Material

Exploring the RNase A scaffold to combine catalytic and antimicrobial activities. Structural characterization of RNase 3/1 chimeras.

Pablo Fernández-Millán, Sergi Vázquez-Monteagudo, Ester Boix* & Guillem Prats-Ejarque*

Faculty of Biosciences, Department of Biochemistry and Molecular Biology, Universitat Autònoma de Barcelona, Barcelona, Spain


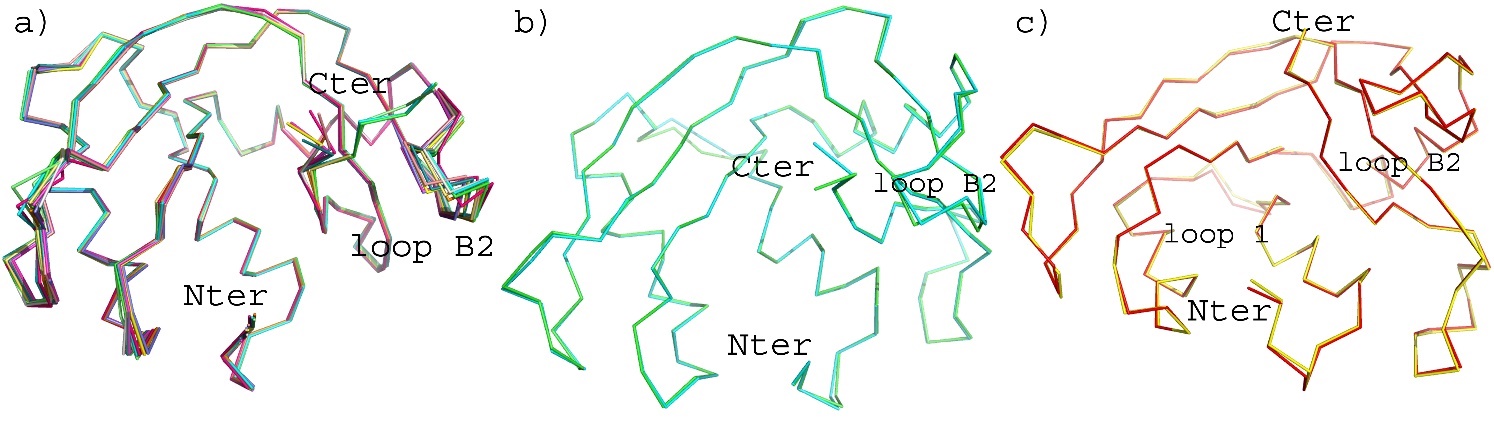


**Figure S1**. Superposition of asymmetric unit backbone chains in RNase 3/1 chimera: a) twelve molecules in RNase 3/1-v1 b) superposition of two crystal structure of version 2 and c) 2 molecules in RNase 3/1-v3.

**A**

**
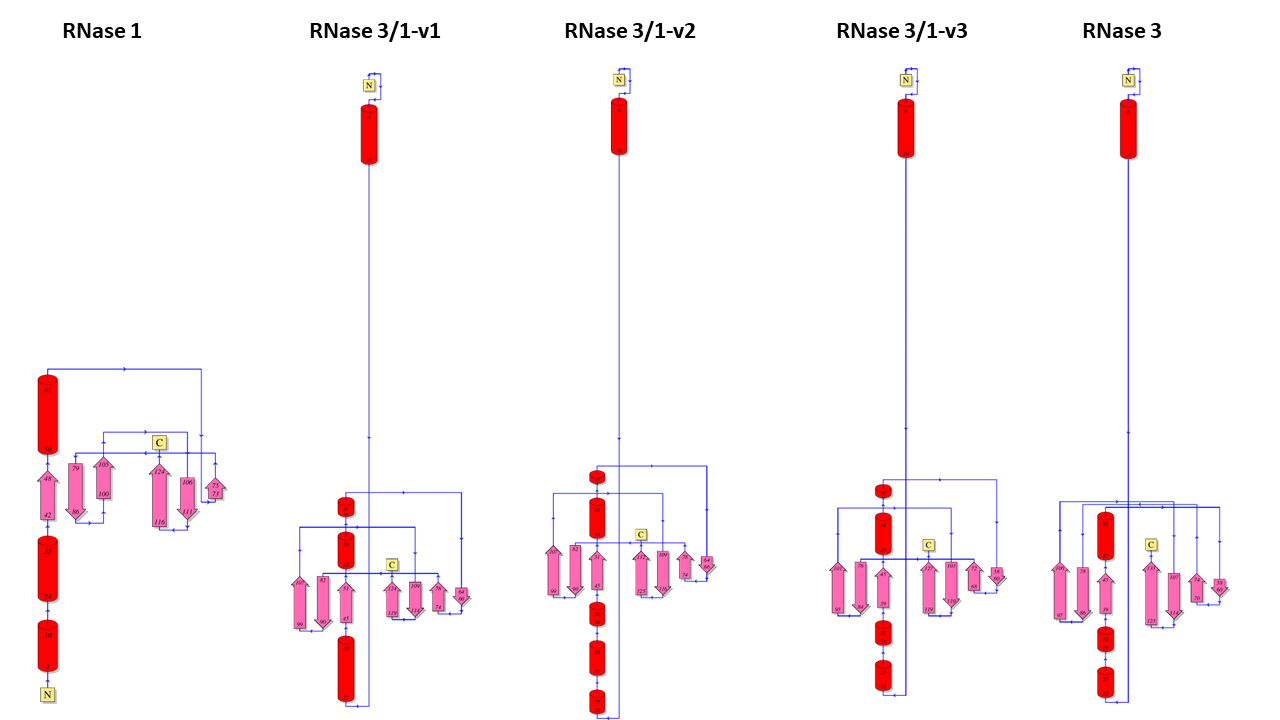
**

**B**

**
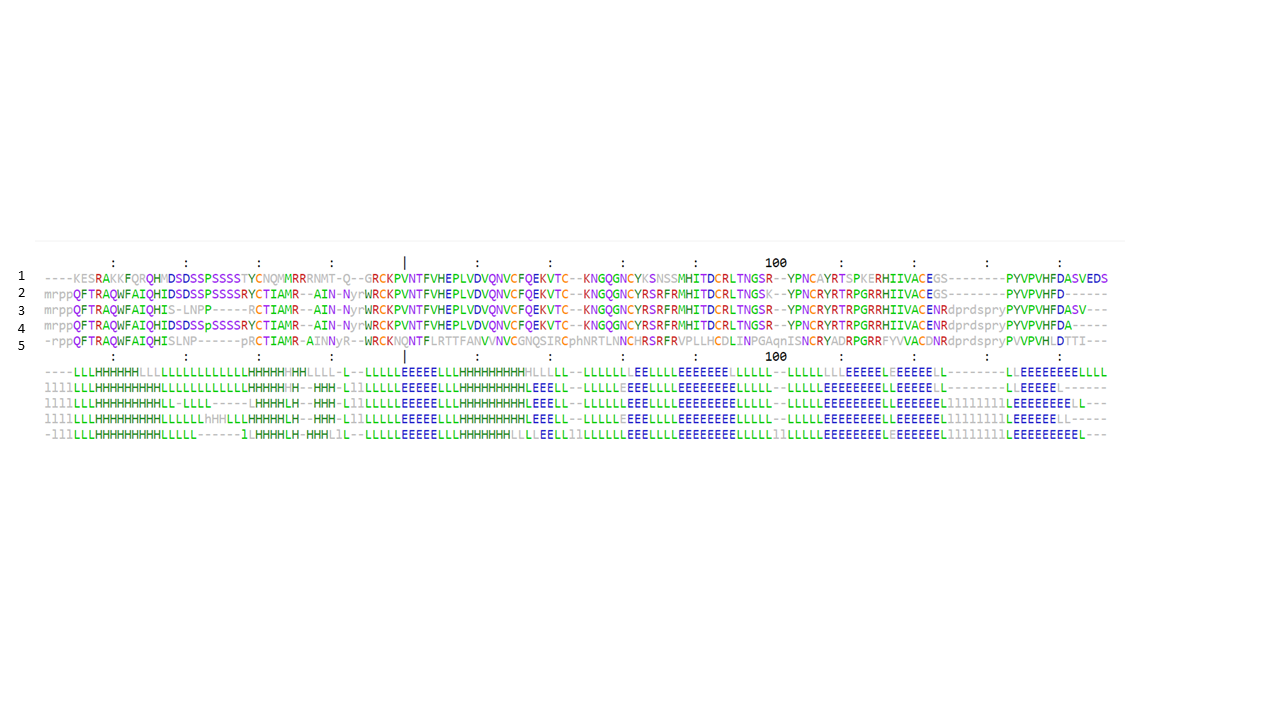
**

**Figure S2**. Structural comparison of RNase 3/1 chimeras with parental RNases 1 and 3. A) Topological models created using *PDBsum*. B) Pairwise secondary structure alignment of RNase 1 (1), RNase 3/1-v1 (2), RNase 3/1-v2 (3), RNase 3/1-v3 (4) and RNase 3 (5) using the *DALI* Server (Holm, *Protein Science* 29: 128, 2020). Secondary elements identified by DSSP are indicated (H, helix, E, strand and L, coil).


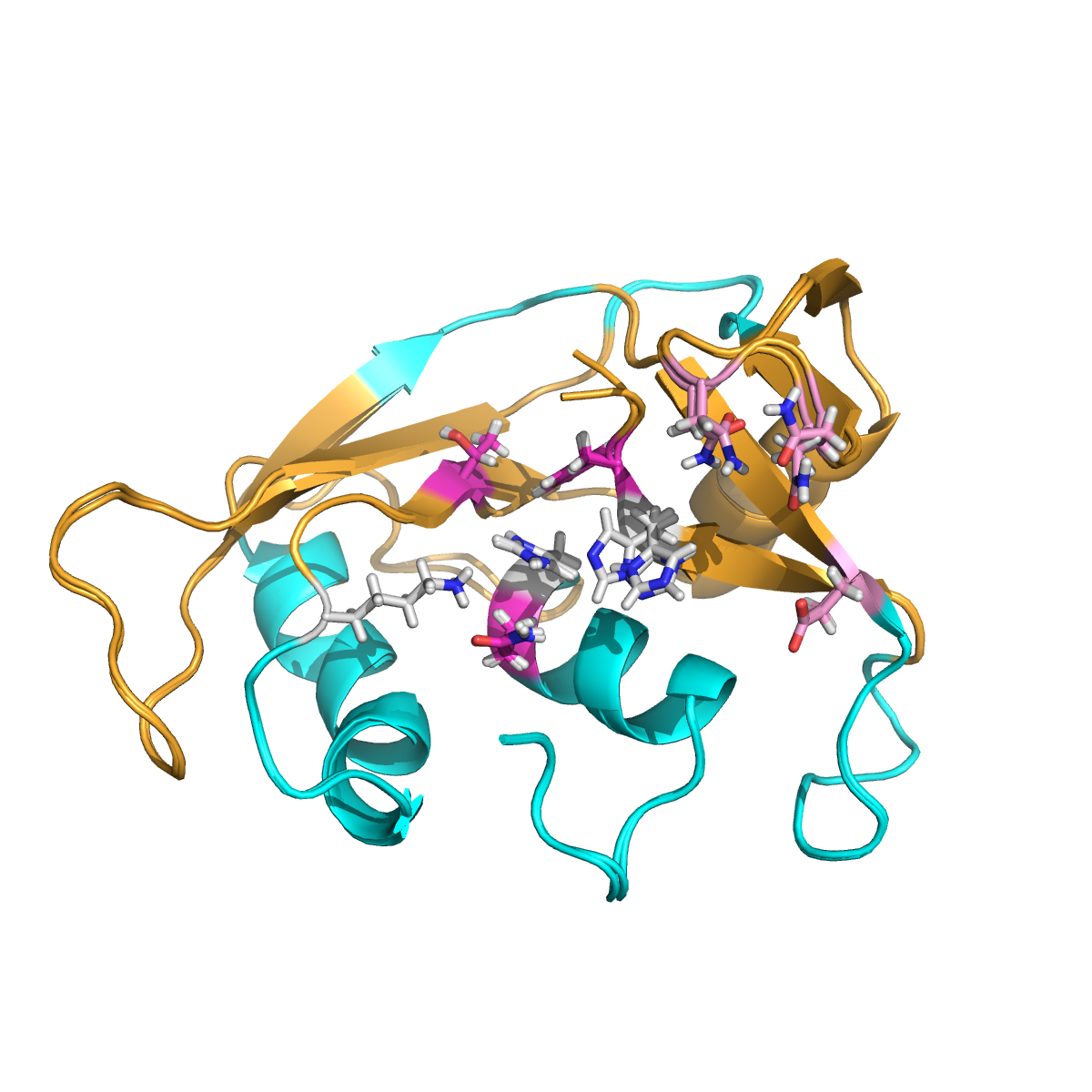


**Figure S3**. Overlapping of the two crystal structures of RNase 3/1-v2 in the absence (6YBE.pdb) and presence of phosphate anions (6YBC.pdb). In light blue, the fragments of RNase 3, in orange, the RNase 1 skeleton. No significant differences can be observed between both structures. Residues involved in catalytic triad, B1 and B2 interactions are colored in grey, pink and magenta respectively. Picture was done by *PyMol*.


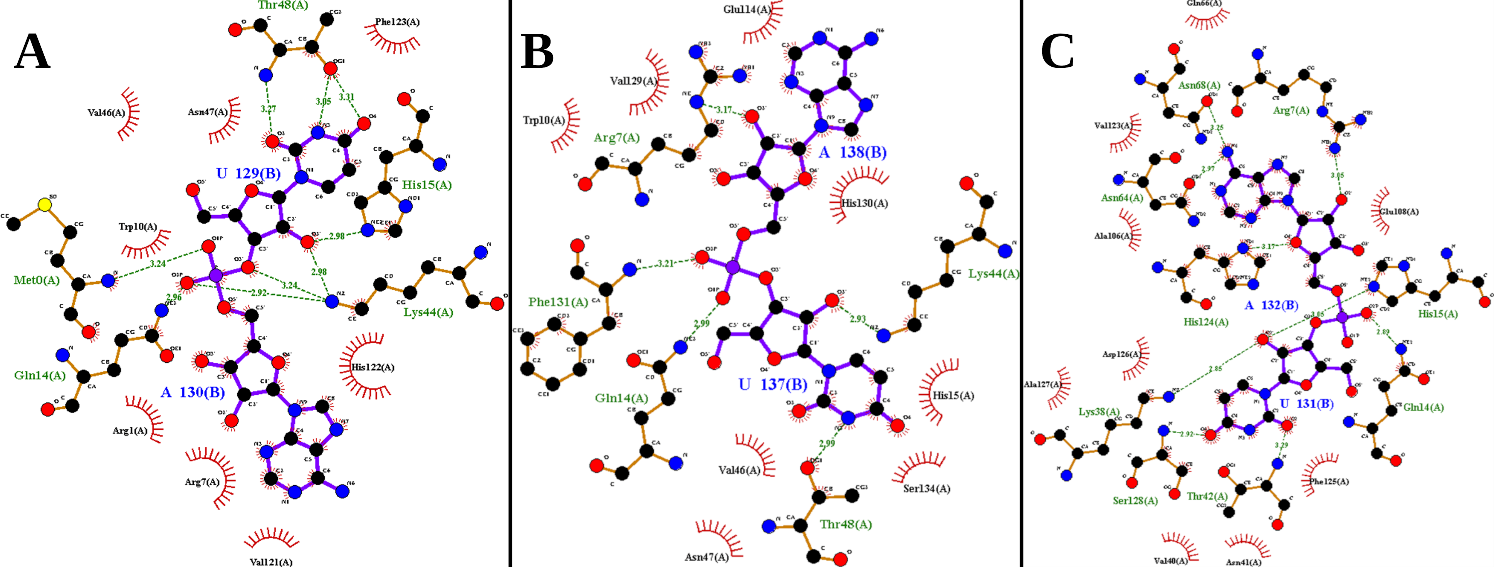


**Figure S4**. Side-by-side comparison of interacting residues between UpA and **A)** RNase 3/1-v1, **B)** RNase 3/1-v2 and **C)** RNase 3/1-v3 in predicted complexes. Figure was done using *LigPlot+* (Laskowski and Swindells, *J. Chem. Inf. Model.* 51:2778, 2011)*.*


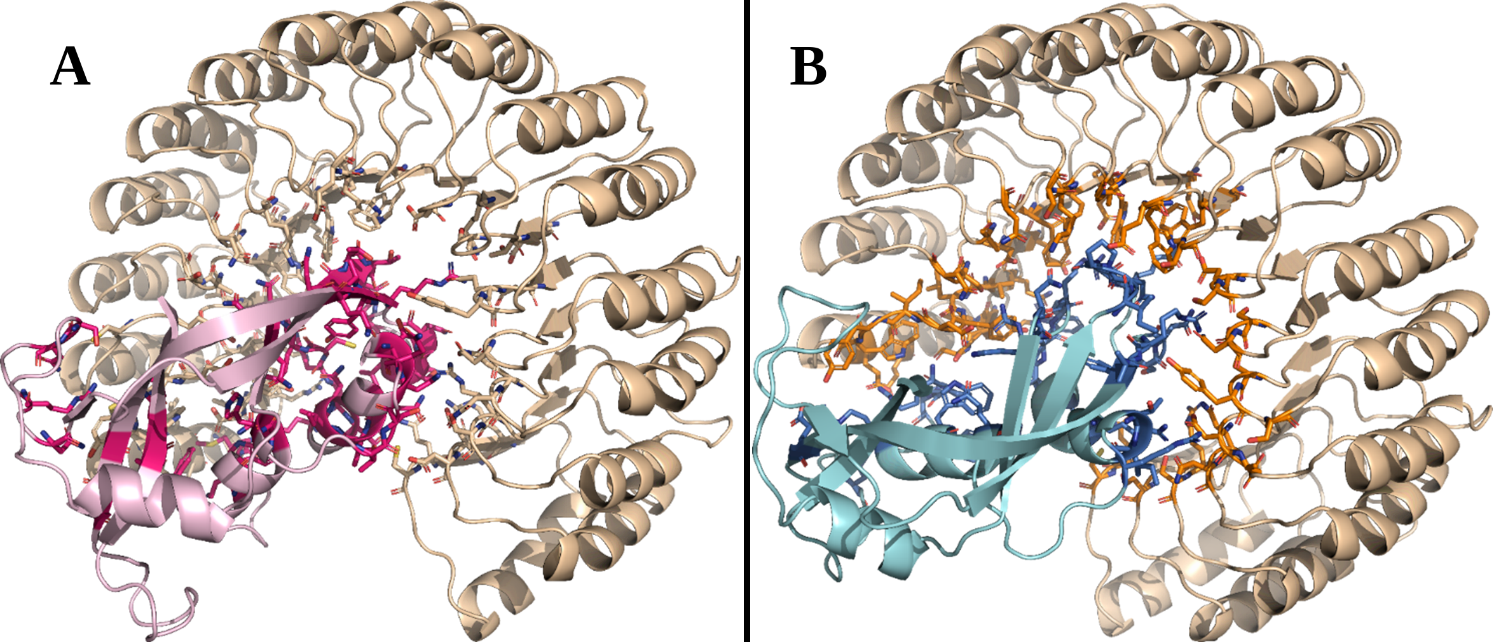


**Figure S5**. Comparison of A) RI-EDN complex (2BEX.pdb) and B) predicted RI-ECP complex using *ClusPro* (Desta et al., *Structure* 28:1071, 2020).

**A**


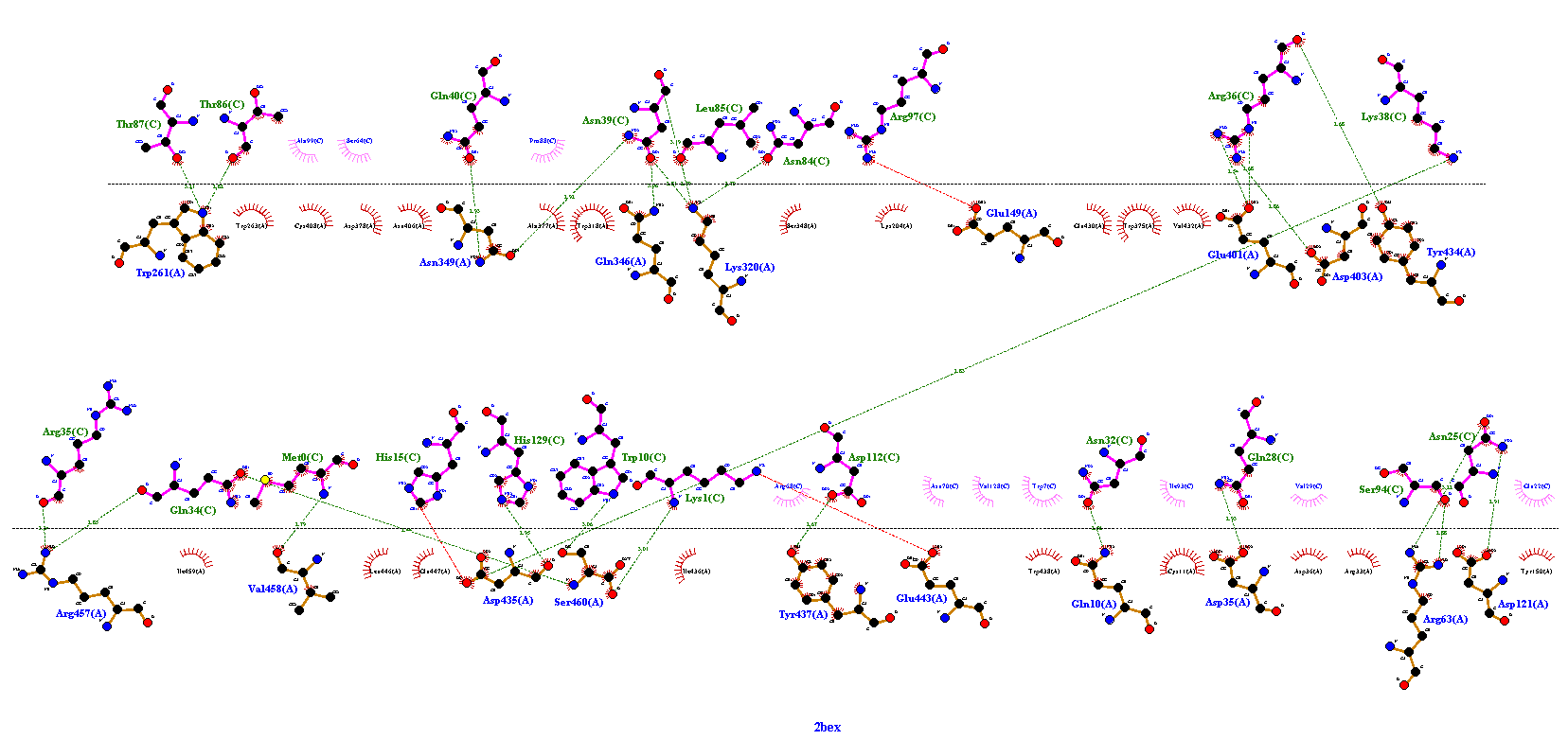


**B**


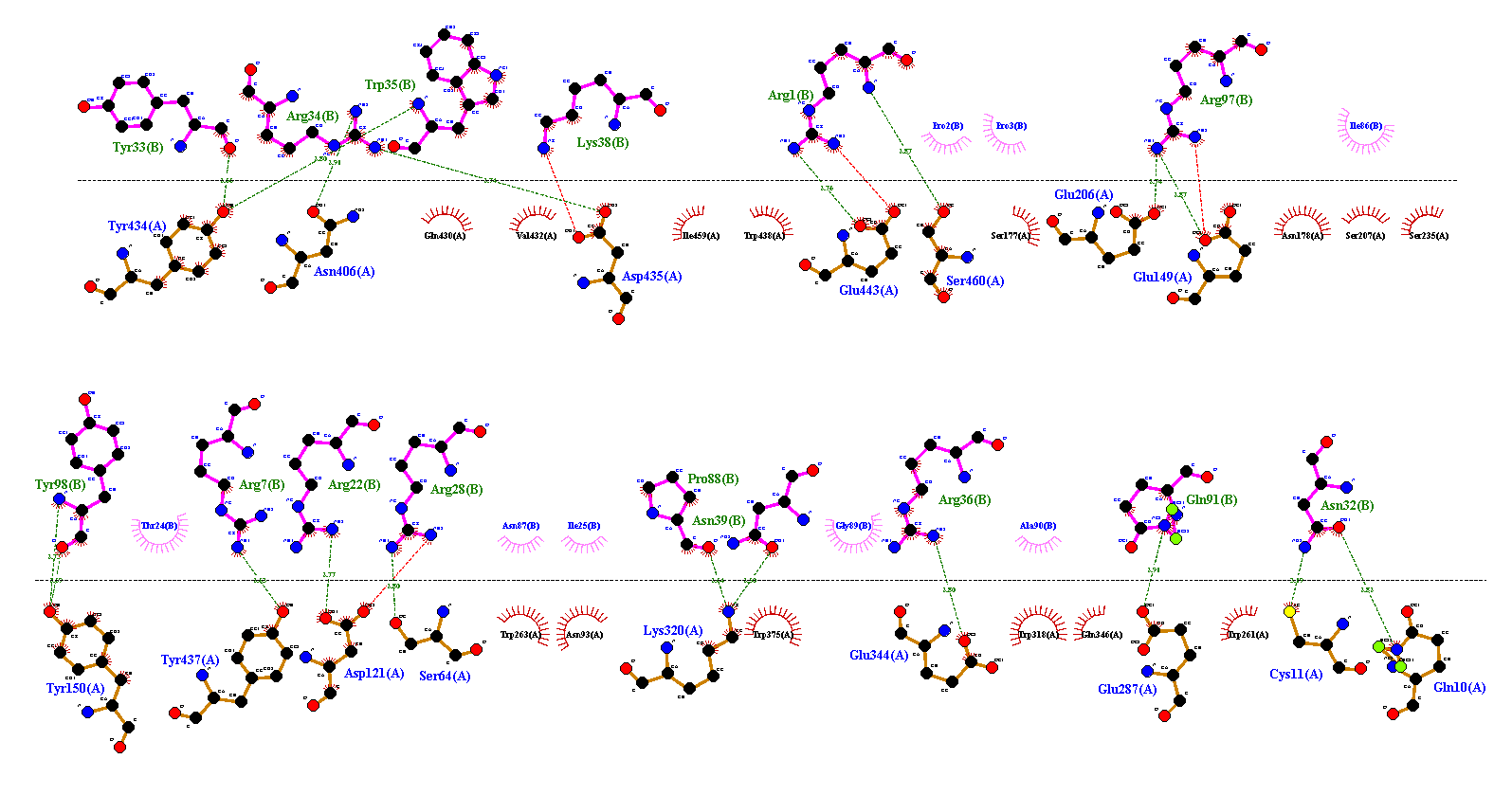


**Figure S6**. Analysis using LigPlot+ of A) RNase 2-RI complex (2BEX.pdb) and B) the predicted RNase 3-RI complex by *ClusPro*. See a list of interacting residues in Annex1 file.

|  | **RNase 3/1-v1**  (6YMT.pdb) | **RNase 3/1-v2**  (6YBE.pdb) | **RNase 3/1-v2 + PO_4_**  (6YBC.pdb) | **RNase 3/1-v3 + PO_4_**  (6SSN.pdb) |
| --- | --- | --- | --- | --- |
| Resolution (Å) | 71.19 - 1.58 (1.64 - 1.58) | 38.47 - 1.12  (1.14 - 1.12) | 38.58 - 1.48  (1.62 - 1.48) | 35.23 - 1.51   - 1. - 1.51) |
| Space group | C 222_1_ | I121 | I121 | P1 |
| Unit cell a, b, c (Å) α, β, γ (o) | 106.84 115.67 284.74 90 90 90 | 43.21, 61.7, 51.35  90, 106.883, 90 | 43.16, 61.74, 51.52  90, 106.461, 90 | 38.38, 39.54, 46.33  73.61, 83.11, 66.64 |
| Total reflections | 477787 (47456) | 90327 (4758) | 65492 (3360) | 63865 (6336) |
| Unique reflections | 239337 (23748) | 45629 (2247) | 21301 (1062) | 31951 (3156) |
| Multiplicity | 2.0 (2.0) | 2.1 | 3.1 | 2.0 |
| Completeness (%) | 99.93 (99.95) | 92.84 (90.6) | 99.4 (99.4) | - 1. (83.59) |
| Mean I/σ(I) | 13.04 (1.83) | 16.9 (14.7) | 9.8 (3.4) | - 1. (1.59) |
| Wilson B-factor | 21.24 | 10.59 | 14.21 | 15.63 |
| R_merge_ | 0.029 (0.46) | 0.029 (0.5) | 0.057 (0.39) | 0.078 (0.44) |
| CC_1/2_ | 0.999 (0.61) | 0.996 (0.97) | 0.996 (0.83) | 0.994 (0.793) |
| Reflections used in refinement | 239313 (23743) | 45652 (4465) | 21296 (2145) | 31945 (3149) |
| Reflections for R_free_ | 11869 (1152) | 1999 (196) | 1999 (202) | 1569 (149) |
| R_work_ | 0.18 (0.29) | 0.14 (0.13) | 0.14 (0.16) | 0.19 (0.27) |
| R_free_ | 0.20 (0.32) | 0.15 (0.18) | 0.17 (0.25) | 0.22 (0.30) |
| Nº of non-H atoms | 14825 | 1391 | 1396 | 2540 |
| Protein | 12856 | 1198 | 1217 | 2214 |
| Ligands | 6 | 0 | 46 | 46 |
| Solvent | 1963 | 179 | 147 | 280 |
| Prot. residues | 1514 | 134 | 135 | 261 |
| RMS (bonds) | 0.009 | 0.010 | 0.009 | 0.008 |
| RMS (angles) | 1.06 | 1.177 | 0.996 | 1.20 |
| Ramachandran favored (%) | 97.92 | 97.74 | 96.85 | 98.05 |
| Ramachandran allowed (%) | 2.08 | 2.26 | 2.39 | 1.95 |
| Ramachandran outliers (%) | 0 | 0 | 0.76 | 0 |
| Rotamer outliers (%) | 0.97 | 0 | 0 | 0 |
| Clashscore | 5.49 | 11.5 | 6.71 | 4.32 |
| Average B-factor | 31.14 | 17.54 | 26.83 | 21.37 |
| Protein | 29.88 | 15.24 | 25.11 | 19.72 |
| Ligands | 35.56 | 17.01 | 43.48 | 32.19 |
| Solvent | 39.41 | 0 | 32.34 | 30.90 |

**Table S1**. Data collection and processing statistics. Statistics for the highest-resolution shell are shown in parentheses.

| **Residues** | **Parental RNase** | **Ascribed role** |
| --- | --- | --- |
| R1 – I16 | RNase 3 | ECP N-terminal, antimicrobial activity (includes aggregation prone region) |
| D17 – S26 | RNase 1 | L1 Flexible loop (related to enhanced catalytic efficiency) |
| R27 | RNase 3 | ECP N-terminal, antimicrobial activity |
| Y28 | RNase 1 | RNase 1 skeleton |
| C29 – K44 | RNase 3 | ECP N-terminal, antimicrobial activity (includes LPS binding region) |
| P45 – Y76 | RNase 1 | Main and secondary base binding site (B1 and B2).  RNase 1 skeleton. |
| R77 – R81 | RNase 3 | Antimicrobial activity (exposed cationic residues) |
| M82 – C98 | RNase 1 | RNase 1 skeleton |
| R99 | - | Conservation of an exposed arginine involved in the bactericidal activity |
| Y100 – T102 | RNase 1 | RNase 1 skeleton |
| R103 – R107 | RNase 3 | Antimicrobial activity (exposed cationic residues) |
| H108 – D127 | RNase 1 | RNase 1 skeleton, related to B1 (Glu111). C-terminal end pruned to remove the last 4 amino acid-tail of RNase 1 |

**Table S2**. Summary of the regions incorporated in the design of the original RNase 3/1 chimera (RNase 3/1-v1).

|  | Ch B | Ch C | Ch D | Ch E | Ch F | Ch G | Ch H | Ch I | Ch J | Ch K | Ch L |
| --- | --- | --- | --- | --- | --- | --- | --- | --- | --- | --- | --- |
| Ch A | 0.389 | 0.650 | 0.538 | 0.686 | 0.383 | 0.621 | 0.386 | 0.308 | 0.515 | 0.517 | 0.237 |
| Aa | 128 | 125 | 125 | 126 | 125 | 125 | 125 | 128 | 125 | 124 | 125 |

**Table S3**. RMSD calculation between all the chains of the asymmetric unit of RNase 3/1 -v1. Residues aligned automatically by LSQMAN are indicated (Aa).

| RMSD | RNase3/1-v2+PO_4_ | RNase3/1-v2 | RNase3/1-v3 |
| --- | --- | --- | --- |
| RNase3/1-v1 | 1.088 | 1.135 | 1.007 |
| RNase3/1-v2+PO_4_ | ---- | 0.410 | 0.606 |
| RNase3/1-v2 | 0.410 | ---- | 0.571 |

**Table S4**. RMSD calculation between the different crystals obtained of RNase 3/1 versions, taking the first chain of the asymmetric unit as a reference.

| **HADDOCK interaction energies (kcal/mol)** | | | | |
| --- | --- | --- | --- | --- |
|  | | **RNase 3/1-v1** | **RNase 3/1-v2** | **RNase 3/1-v3** |
| **CpA** | Van der Waals (VdW) | -22.0 +/- 1.7 | -27.2 +/- 2.2 | -35.9 +/- 1.4 |
|  | Electrostatic | -91.2 +/- 12.8 | -74.5 +/- 20.8 | -68.0 +/- 10.4 |
|  | Desolvation | -2.3 +/- 1.0 | -2.4 +/- 0.8 | 1.8 +/- 1.5 |
| **CpG** | Van der Waals (VdW) | -28.8 +/- 2.2 | -32.6 +/- 3.5 | -38.6 +/- 2.0 |
|  | Electrostatic | -71.8 +/- 8.1 | -69.5 +/- 11.9 | -65.8 +/- 9.4 |
|  | Desolvation | -2.5 +/- 1.6 | -0.4 +/- 0.6 | 3.2 +/- 0.8 |
| **UpA** | Van der Waals (VdW) | -26.7 +/- 1.6 | -24.0 +/- 3.8 | -34.6 +/- 1.5 |
|  | Electrostatic | -91.0 +/- 16.5 | -124.3 +/- 27.7 | -83.7 +/- 5.3 |
|  | Desolvation | -2.8 +/- 2.8 | -2.9 +/- 1.3 | 2.6 +/- 0.3 |
| **UpG** | Van der Waals (VdW) | -29.0 +/- 3.4 | -30.2 +/- 2.3 | -37.5 +/- 3.2 |
|  | Electrostatic | -66.2 +/- 20.2 | -78.1 +/- 9.9 | -79.9 +/- 12.2 |
|  | Desolvation | -0.8 +/- 0.9 | -0.6 +/- 1.2 | 3.9 +/- 0.6 |

**Table S5**. Van der Waals, electrostatic and desolvation interaction energies calculated by HADDOCK software for CpA, UpA, CpG and UpG in complex with solved RNases 3/1-v1, -v2 and -v3 structures.

|  | **RNase1** | **RNase3/1-v1** | | **RNase3/1-v2** | | | **RNase3/1-v3** | **RNase3** |
| --- | --- | --- | --- | --- | --- | --- | --- | --- |
| No. of intermolecular contacts | 95 | 112 | | 146 | | | 95 | 103 |
| No. of charged-charged contacts | 14 | 18 | | 21 | | | 10 | 14 |
| No. of charged-polar contacts | 19 | 29 | | 25 | | | 23 | 21 |
| No. of charged-apolar contacts | 24 | 24 | | 42 | | | 22 | 25 |
| No. of polar-polar contacts | 5 | 7 | | 7 | | | 9 | 6 |
| No. of apolar-polar contacts | 21 | 23 | | 27 | | | 17 | 19 |
| No. of apolar-apolar contacts | 12 | 11 | | 24 | | | 14 | 18 |
| Percentage of apolar NIS residues | 30.13 | 32.89 | | 33.51 | | | 32.99 | 31.76 |
| Percentage of charged NIS residues | 32.41 | 32.63 | | 32.97 | | | 33.51 | 34.12 |
| Predicted binding affinity (kcal.mol-1) | -13,3 | -13,2 | | -16,1 | | | -10,4 | -12,1 |
| Predicted dissociation constant (M) at 25˚C | 1.6e-10 | | 1.9e-10 | | 1.6e-12 | 2.5e-08 | | 1.2e-09 |

**Table S6**. Estimated parameters by Prodigy for RNases-RNHI complexes predicted by *ClusPro*.
